# Tegument Glycoproteins and Cathepsins of Newly Excysted Juvenile *Fasciola hepatica* Carry Mannosidic and Paucimannosidic *N*-glycans

**DOI:** 10.1371/journal.pntd.0004688

**Published:** 2016-05-03

**Authors:** Andres Garcia-Campos, Alessandra Ravidà, D. Linh Nguyen, Krystyna Cwiklinski, John P. Dalton, Cornelis H. Hokke, Sandra O’Neill, Grace Mulcahy

**Affiliations:** 1 School of Veterinary Medicine, Veterinary Sciences Centre, University College Dublin, Dublin, Ireland; 2 Conway Institute of Biomedical and Biomolecular Research, University College Dublin, Dublin, Ireland; 3 School of Biotechnology, Faculty of Science and Health, Dublin City University, Dublin, Ireland; 4 Department of Parasitology, Leiden University Medical Center, Leiden, The Netherlands; 5 School of Biological Sciences, Medical Biology Centre (MBC), Queen’s University of Belfast, Belfast, Northern Ireland; University of Melbourne, AUSTRALIA

## Abstract

Recently, the prevalence of *Fasciola hepatica* in some areas has increased considerably and the availability of a vaccine to protect livestock from infection would represent a major advance in tools available for controlling this disease. To date, most vaccine-target discovery research on this parasite has concentrated on proteomic and transcriptomic approaches whereas little work has been carried out on glycosylation. As the *F*. *hepatica* tegument (Teg) may contain glycans potentially relevant to vaccine development and the Newly Excysted Juvenile (NEJ) is the first lifecycle stage in contact with the definitive host, our work has focused on assessing the glycosylation of the NEJTeg and identifying the NEJTeg glycoprotein repertoire. After *in vitro* excystation, NEJ were fixed and NEJTeg was extracted. Matrix-assisted laser desorption ionisation-time of flight-mass spectrometry (MALDI-TOF-MS) analysis of released *N-*glycans revealed that oligomannose and core-fucosylated truncated *N-*glycans were the most dominant glycan types. By lectin binding studies these glycans were identified mainly on the NEJ surface, together with the oral and ventral suckers. NEJTeg glycoproteins were affinity purified after targeted biotinylation of the glycans and identified using liquid chromatography and tandem mass spectrometry (LC-MS/MS). From the total set of proteins previously identified in NEJTeg, eighteen were also detected in the glycosylated fraction, including the *F*. *hepatica* Cathepsin B3 (FhCB3) and two of the Cathepsin L3 (FhCL3) proteins, among others. To confirm glycosylation of cathepsins, analysis at the glycopeptide level by LC-ESI-ion-trap-MS/MS with collision-induced dissociation (CID) and electron-transfer dissociation (ETD) was carried out. We established that cathepsin B1 (FhCB1) on position N80, and FhCL3 (BN1106_s10139B000014, scaffold10139) on position N153, carry unusual paucimannosidic Man_2_GlcNAc_2_ glycans. To our knowledge, this is the first description of *F*. *hepatica* NEJ glycosylation and the first report of *N-*glycosylation of *F*. *hepatica* cathepsins. The significance of these findings for immunological studies and vaccine development is discussed.

## Author Summary

*Fasciola hepatica* is a parasite responsible for the zoonotic disease fasciolosis, prevalence of which has increased in recent years because of the emergence of triclabendazole-resistant strains as well as changing climatic conditions. A number of *F*. *hepatica* protein antigens are used for assessing the immune response of the definitive host for the development of recombinant vaccines but no such vaccine has been commercialised yet. Glycans that may contribute to the antigenic, immunological and protective properties in *F*. *hepatica* have not been characterised. Using a panel of plant lectins with defined sugar binding specificities alongside mass spectrometric analysis, we found that high mannose and oligomannose *N-*glycans are the most abundant in the surface of the juvenile fluke and identified eighteen proteins likely to be glycosylated. Additionally, we found that the proteases cathepsin B1 and L3 contain a glycosylation site occupied by an unusually short Man_2_GlcNAc_2_ *N-*glycan. This work is the starting point for understanding how *F*. *hepatica* glycans interact with the definitive host at the initiation of infection. Additionally, it provides useful information for including glycans in the design of new vaccine candidates.

## Introduction

The trematode *Fasciola hepatica*, commonly known as the liver fluke, is widely distributed across five continents and is responsible for fasciolosis in livestock and humans. It has a large economic impact on the livestock industry, causing production losses in terms of reduced milk yield, liver condemnation and problems with animal fertility among others [1–3]. The World Health Organisation (WHO) has estimated an increase of 11% in the prevalence of human fasciolosis over the last decade, with more than 2.6 million people infected globally [4]. To date, the chief method of control has been administration of flukicidal drugs, with triclabendazole the most frequently used. However, in recent decades, triclabendazole resistance in fluke populations has been reported globally [5]. This, together with the goal of reducing drug use in food-producing animals [6] has encouraged the scientific community to investigate new methods of control. The development and use of vaccines against *F*. *hepatica* would be a more sustainable and environmentally friendly future alternative to anthelmintic drugs.

The tegument (Teg) and the excretory/secretory (ES) components are important sources of *F*. *hepatica* antigens with the highest potential as vaccine targets. Native *F*. *hepatica* molecules identified and isolated using proteomic approaches have in some cases been able to produce significant reductions in liver fluke burden and reduced pathology not only in small animal models but also in cattle and sheep. For example, the native members of the Cathepsin clade FhCL1, FhCL2 and FhCL3—some of the most intensively studied vaccine candidates—were able to induce protection in experimental models including rats [7], and in cattle [8] and sheep [9]. In addition, they were able to decrease liver fluke egg viability by as much as 98% in vaccinated animals [8,10]. Other important vaccine candidates investigated included peroxiredoxin (PRX), paramyosin, glutathione S-transferase (GST), fatty acid-binding protein [11] and leucine-aminopeptidase [9]. In many cases, the protective capacity of recombinant versions was lower than that of the native proteins, or more variable between one study and another. For example, the recombinant version of FhCL1 induced 48% protection in cattle [12] in a small-scale field trial but did not provide protection in small ruminants [13,14]. Although animal variability and differences in vaccine formulation are key factors that could explain these discrepancies [15], it is also likely that there are differences in terms of protein folding or post-translational modifications between native and recombinant proteins which influence protective capacity. Glycosylation is one of the main post-translational modifications that occurs following protein synthesis, and glycosylation pathways of prokaryotic and simple eukaryotic vectors used to produce recombinant vaccine candidates are substantially different from those of the parasite itself [16].

Glycomic studies of other trematodes, such as *Schistosoma mansoni* [17] and *Opisthorchis viverrini* [18] and most recently *Echinostoma caproni* [19,20] have led to a deeper understanding of the structural and functional aspects of glycans in this class of helminths. The immunomodulatory properties of *F*. *hepatica* glycans and glycoconjugates are now also being studied; for example, their apoptotic effect in peritoneal eosinophils and macrophages *in vitro* has been demonstrated [21,22] along with their induction of arginase 1, IL-10 and TGF-β transcription in peritoneal macrophages, indicators of M2a macrophages [23]. Recently, the requirement of *F*. *hepatica* glycans to influence dendritic cell (DC) maturation and to inhibit IFN-γ production by splenocytes from infected animals has been reported [24,25].

It has been shown that the adult stage of *F*. *hepatica* contains at least several types of glycans and glycan motifs such as fucosylated LacdiNAc (LDN-F) motifs [26], mucin-type *O-*glycosylated proteins [27], the glycolipid CD77 [28] and other glycoceramides [29,30]. Even though the full range of *F*. *hepatica* glycoconjugates has not been characterised, the presence of various glycans has been confirmed by lectin binding studies not only in adult stages [25,29,31] but also in the other lifecycle stages (miracidia, rediae, sporocysts) [32–34]. However, systematic studies of glycosylation in the NEJ have not yet been carried out. It is also important to characterise the protein backbones containing these glycans and to verify whether proteins used previously as vaccine candidates are glycosylated. Finally, the potential immunomodulatory properties of these glycans are important both for our understanding of the immunology of fasciolosis and for vaccine development.

The goals of this work were to describe the glycosylation patterns of NEJ tegumental (NEJTeg) and somatic (NEJSom) fractions using lectin-affinity techniques and perform a deeper glycan characterisation of NEJTeg using mass spectrometry (MS). We also identified NEJ proteins that are glycosylated and assessed whether they have been previously described as antigenic/vaccine candidates.

## Materials and Methods

### *In vitro* excystation of metacercariae and isolation of NEJTeg and NEJSom

*F*. *hepatica* metacercariae with their outer cyst wall removed were obtained from Baldwin Aquatics, Inc. (Monmouth, Oregon) and stored at 4°C until use. Excystation of metacercariae was performed as described previously with minor modifications [35]. Approximately 15,000 excysted NEJ were used for Teg isolation as previously described [36]. Briefly, NEJ were washed three times in PBS followed by incubation for 30 min at room temperature with 1ml of 1% Nonidet P40 (NP40) in PBS. They were then centrifuged at 300 x g for 5 min and the supernatant collected. NP40 was removed from the supernatant with 0.3 g of Bio beads (Bio-Rad) according to the manufacturer’s recommendations. The pellet obtained after Teg removal, containing denuded NEJ, was suspended in 1 ml of RIPA buffer (Sigma-Aldrich) and used for somatic fraction isolation as previously described [37]. After incubation, both samples were centrifuged at 1000 x g for 5 min and supernatants, which correspond to the NEJTeg and NEJSom, respectively, were aliquoted and stored at -80°C. The protein content was determined by the bicinchoninic acid assay (Thermo Fisher Scientific) according to the manufacturer’s recommendations.

### Lectin fluorescence staining

The protocol for lectin fluorescence staining was adapted from that described [38]. After fixation in 10% buffered paraformaldehyde solution, NEJ were incubated overnight with 10 ml of 1% BSA, 0.1% sodium azide in PBS (blocking solution) at 4°C. Following extensive washing in PBS, they were suspended in 500 μl of 0.1% BSA, 0.1% sodium azide in PBS (ABD buffer) and aliquoted in batches (40 per batch). NEJ were then incubated overnight in the dark at RT with a panel of FITC conjugated-lectins (Vector Labs) diluted in ABD buffer in a ratio at 1:200. The lectins used and their nominal binding specificity are listed in Table 1. Specific lectin binding was confirmed by pre-incubation of each lectin with the appropriate sugar inhibitor for 2 h at the concentration provided in Table 1. After incubation and extensive washes with ABD solution, NEJ were placed in Vectashield mounting medium with DAPI (Vector Labs). Slides were viewed with a LEICA DM IL LED using 10x and 40x HI PLAN I objectives (Leica Microsystems) equipped with an epifluorescence source and filter system for FITC and DAPI fluorescence. Images were merged using Adobe Photoshop CC software version 14.0 x 64.

**Table 1:** Origin and nominal specificity of the lectins used for *F*. *hepatica* NEJTeg and NEJSom lectin blot and lectin fluorescence staining.

Lectin	Origin	Nominal specificity	Specific inhibitors
**AAL**^a^	*Aleuria aurantia*	Fuc (α1–6)GlcNAc and Fuc (α1–3) LacNAc	Fuc 0.2 M
**ConA**^a^	*Canavalia ensiformis*	α-Man; α-Glc	Man 0.5 M + Glc 0.5 M
**LCA**^a^	*Lens culinaris*	α-Man; α-Fuc	Man 0.5 M + Glc 0.5 M
**PSA**^a^	*Pisum sativum*	α-Man; α-Fuc	Man 0.5 M + Glc 0.5 M
**GNL**^a^	*Galanthus nivalis*	(α1–3)Man	Man 0.5 M + Glc 0.5 M
**WGA**^a^	*Triticum vulgaris*	GlcNAc oligomers; NeuAc	GlcNAc 0.5 M
**S-WGA**^a^	*Triticum vulgaris*	GlcNAc	GlcNAc 0.5 M
**GSL II**^a^	*Griffonia simplicifolia*	α- or β-GlcNAc	GlcNAc 0.5 M
**RCA-120**^a^	*Ricinus communis*	Gal (pref β); GalNAc	Gal 0.5 M
**GSL I**^a^	*Griffonia simplicifolia*	α-GalNAc; α-Gal	Gal 0.5 M+ GalNAc 0.5 M
**SBA**^a^	*Glycine max*	α- or β-GalNAc; Gal	GalNAc 0.5 M
**DBA**^a^	*Dolichos biflorus*	α-GalNAc	GalNAc 0.5 M
**PNA**^a^	*Arachis hypogaea*	Gal (β1–3)GalNAc (= T-antigen”) on/or *O*-Glycans	Gal 0.5 M
**PHA-L**^a^	*Phaseolus vulgaris*	Oligosaccharide	Acetic acid 0.1 M
**PHA-E**^a^	*Phaseolus vulgaris*	Oligosaccharide	Acetic acid 0.1 M
**Jacalin**^a^	*Artocarpus integrifolia*	Gal (β1–3)GalNAc (= T-antigen”) on/or *O*-Glycans	Gal 0.5 M
**ECL**^a^	*Erythrina cristagalli*	LacNAc; Gal	Lac 0.5 M
**SJA**	*Sophora japonica*	(pref. β-) GalNAc and Gal	GalNAc 0.2 M
**UEA I**	*Ulex europaeus*	α-Fuc	Fuc 0.1 M
**VVL**	*Vicia villosa*	α- or β-GalNAc (terminal) and α- GalNAc-Ser/Thr (= Tn-antigen) and Gal(α1–3)GalNAc	GalNAc 0.2 M
**DSL**	*Datura stramonium*	(β1–4) GlcNAc oligomers (chitobiose etc); LacNAc	Chitin Hydrolase
**LEL**	*Lycopersicon esculentum*	GlcNAc oligomers	Chitin Hydrolase
**MAL II**	*Maackia amurensis*	(α2–3)NeuAc	Human glycophorin
**SNA**	*Sambucus nigra*	NeuAc(α2–6)Gal > (α2–3); Lac/Gal	Lac 0.5M in buffered saline followed by Lac 0.5M in acetic acid
**STL**	*Solanum tuberosum*	GlcNAc oligomers; bacterial cell walls	Chitin Hydrolase

^a^Lectins with their specific inhibitors for each lectin employed in fluorescence staining: Fuc (Fucose), Man (Mannose), Glc (Glucose), Gal (Galactose), Lac (Lactose), GlcNAc (*N-*Acetyl-Glucosamine), GalNAc (*N-*Acetyl-Galactosamine), LacNAc (*N-*Acetyl-Lactosamine), NeuAc (Neuraminic acid = sialic acid), Ser (serine), Thr (threonine).

### Lectin blot analysis

NEJTeg and NEJSom (10 μg) were analysed by SDS–PAGE using 4–20% gradient Tris-glycine precast gels (Thermo Fisher Scientific) in a vertical electrophoresis system (ATTO) for 90 min at 40 mA. Gels were either subjected to silver staining or transferred to nitrocellulose membrane using the iBlot system (Thermo Fisher Scientific). After blocking with 5% BSA in PBS for 1 h, membranes were incubated with biotin-conjugated lectins (Vector Labs) for 1 h. For secondary detection, membranes were washed and incubated with IRDye-labelled streptavidin (LI-COR Biosciences) for 1 h. Specific lectin binding was confirmed as described previously. A membrane incubated with IRDye-labelled streptavidin only was performed as an additional negative control in order to confirm lack of reactivity between NEJ extracts and streptavidin. Images were acquired using an Odyssey infrared scanning imaging system (LI-COR Biosciences).

### *N-*glycan release, purification, labelling and exoglycosidase treatment

NEJTeg (glyco)proteins (500 μg) were enzymatically fragmented by using trypsin-coupled beads (GE Healthcare Life Sciences) following the manufacturer’s instructions. Four μl of 1 U/μl N-glycosidase-F (PNG-F) (Roche) were added to the sample and incubated for 24 h at 37°C while shaking. The *N-*glycan mixture released by the PNG-F was purified with a C18 Reverse phase (RP) cartridge (500 mg; JT Baker) and a carbon cartridges (150 mg Carbograph; Grace) as previously described [39].

The (glyco)peptides remaining in the C18 RP cartridge were eluted, dried in a Speed-Vac (Thermo Fisher Scientific) and dissolved in 50 μl of 1 M sodium acetate solution (pH 4.5). Following pH adjustment to 4.5, 2 μl of *N-*glycosidase-A (PNG-A, 1 U/μl; Roche) were added to the sample and incubated for 48 h at 37°C while shaking. The *N-*glycan mixture released by the PNG-A treatment was purified and incubated overnight as described previously [39]. The purified *N-*glycans were subsequently labelled with the fluorophore 2-aminobenzoic acid (2-AA) (Sigma-Aldrich), as described elsewhere [40]. The labelled *N-*glycans were brought to 75% AcN and loaded on Biogel P10 (Bio-Rad) columns conditioned with 80% AcN. *N-*glycans were eluted with 400 μl of dH_2_O and dried down in a Speed-Vac. AA-labelled PNG-F released glycans (1 μl/treatment) were incubated in a volume of 10 μl for 24 h at 37°C in 250 mM sodium citrate buffer with one of the following exoglycosidases: α-mannosidase from jack bean (15 μU/μl; Sigma Aldrich), β-galactosidase from jack bean (5 μU/μl; Prozyme) or recombinant β-*N*-acetylglucosaminidase (4 μU/μl; New England Biolabs).

### MS and glycan composition determination

PNG-F and PNG-A released *N-*glycans were dissolved in 50 and 25 μl of dH_2_O respectively. One μl of each sample was spotted on a matrix-assisted laser desorption ionisation (MALDI) polished steel targeted plate (Bruker Daltonics) using 2,5-dihydroxybenzoic acid (DHB; 20 mg/ml in 30% AcN) as matrix. Samples were analysed with an Ultraflex II MALDI–time-of-flight (MALDI-TOF) mass spectrometer (Bruker Daltonics, Bremen, Germany) operating in the negative-ion reflectron mode. For assessing the results of exoglycosidase treatments, digestion products were purified using a HILIC Ziptip column (Millipore) following the manufacturer’s instructions. Glycans were eluted directly to a MALDI target plate with 10 mg/ml DHB in 50% AcN containing 0.1% TFA and analysed in the negative-ion reflectron mode. Glycopeakfinder (http://www.glyco-peakfinder.org) was used to define glycan composition.

### NEJTeg glycoprotein biotinylation and isolation

The glycan components of NEJTeg-derived glycoproteins were biotinylated with EZ-link Hydrazide-Biotin (Thermo Fisher Scientific) according to the manufacturer’s instructions. The biotinylated glycoproteins (B-NEJTeg) were purified from the rest of the NEJTeg components by affinity chromatography using monomeric avidin agarose (Thermo Fisher Scientific). The flow through was collected as avidin-unbound fraction (A-UB-NEJTeg) and the avidin-bound fraction (A-BB-NEJTeg) separated from the resin following a previously reported protocol [41]. All fractions were concentrated and buffer exchanged in PBS using Pierce Concentrator 3K MWCO (Thermo Fisher Scientific). BCA assays were performed in order to quantify the protein content. SDS–PAGE, silver staining and streptavidin blots were used in order to assess the quality of the biotinylation reaction.

### Glycoprotein identification

Total NEJTeg (50 μg), B-NEJTeg and A-BB-NEJTeg, samples (10 μg each) were fractionated by 12% SDS-PAGE. The bands contained in B-NEJTeg and A-BB-NEJTeg fractions were stained with SYPRO Ruby gel stain (Bio-Rad) according to the manufacturer’s instructions. Bands were excised and a liquid handling station (MassPrep; Waters) was used with sequencing-grade modified trypsin (Promega) according to the manufacturer’s instructions, for in-gel protein digestion. Peptide extracts were then dried by evaporation in a Speed-Vac. Liquid Chromatography and tandem mass spectrometry (LC-MS/MS) was carried out with a linear trap quadrupole (LTQ) mass spectrometer connected to a Thermo Surveyor MS pump and equipped with a nano electrospray ionisation (ESI) source (Thermo Fisher Scientific) [42].

For total NEJTeg, bands were stained with a Coomassie blue kit (Thermo Fisher Scientific), excised and digested as described [43]. The proteomics analysis of the NEJTeg extract was performed by nano reverse-phase (RP) LC-ESI-ion trap MS/MS, consisting of an Ultimate 3000 RSLC nano LC system (Thermo Fisher Scientific) coupled to a Captive Spray nano Booster (Bruker Daltonics) according to previous protocol [43].

All MS/MS spectra were analysed with Mascot (version 2.4.0; Matrix Science), set up to search against two proprietary *Fasciola hepatica* databases, assuming digestion with trypsin with 1 miss cleavage allowed: (1) a database comprising the gene models identified from the *F*. *hepatica* genome (101,780; [44]) (2) a database comprising all available EST sequences (633,678 entries). Fragment and parent ion mass tolerance were set at 0.100 Da. Carbamidomethylation of cysteine was specified as a fixed modification. Dehydration of the N-terminus, Glu->pyro-Glu of the N-terminus, ammonia-loss of the N-terminus, Gln->pyro-Glu of the N-terminus, deamidation of Asn and Glu, oxidation of Met, Arg and Thr and biotinylation of Lys were specified as variable modifications. Scaffold v4.34 (Proteome Software Inc.) was used to validate MS/MS based peptide and protein identifications. Peptide identifications were accepted if they could be established at greater than 95% probability to achieve a 0% false discovery rate (FDR) by the Peptide Prophet algorithm [45] with Scaffold delta-mass correction. Protein identifications were accepted if they could be established at greater than 95% probability to achieve a 0% FDR and contained at least 2 identified peptides. Protein probabilities were assigned by the Protein Prophet algorithm [46]. Proteins that contained similar peptides and could not be differentiated based on MS/MS analysis alone were grouped to satisfy the principles of parsimony. Putative *N-*glycosylation sites of the glycoproteins detected in the A-BB-NEJTeg and from the cathepsins identified in total NEJTeg were searched using the NetNGlyc 1.0 Server (www.cbs.dtu.dk) [47].

### Prediction of tryptic peptide of cathepsins with putative *N*-glycosylation and in-depth glycopeptide MS analysis

Bands from the NEJTeg sample in which the presence of cathepsins were confirmed by MS/MS were selected for in-depth glycopeptide analysis. The amino acid sequence and the molecular weight of the *N-*glycosylated tryptic peptides of the cathepsins were predicted using the online tool http://web.expasy.org/peptide_mass [48] taking into account carbamidomethylation of cysteine, and methionine oxidation as a variable modification.

For nano-RP-LC-ESI-ion trap-MS/MS the peptides and glycopeptides extracted from the cathepsin bands were loaded on a trap column (Acclaim PepMap100 C18 column, 100 μm × 2 cm, C18 particle size 5 μm, pore size 100 Å, Thermo Fisher Scientific) for concentration prior to separation on an Acclaim PepMap RSLCnano-column (75 μm × 15 cm, C18 particle size 2 μm, pore size 100 Å, Thermo Fisher Scientific). The column was equilibrated at RT with eluent A (0.1% formic acid in dH_2_O) at a flow rate of 700 nL/min. After injection of the sample, elution conditions were switched to 10% solvent B (95% AcN, 5% dH_2_O), followed by a gradient to 60% B in 45 min and a subsequent isocratic elution of 10 min. The eluate was monitored by absorption at 215 nm. MS was performed on an AmazonSpeed ion trap (Bruker Daltonics) containing an electron-transfer dissociation (ETD) module (PTM Discovery System). The MS instrument was operated in positive ion mode with a mass window of *m/z* 400–2000. The five most abundant ions in an MS spectrum were selected for MS/MS analysis by collision-induced dissociation (CID) using helium as the collision gas, with ion detection over *m/z* 300–1300. For the electrospray (1300 V), solvent evaporation was achieved at 180°C with N_2_ stream at a flow rate of 3 L/min.

In manually selected cases glycopeptide sequence analysis was performed using ETD as described [49]. The selected glycopeptide ions were isolated in the ion trap and fluoranthene radical anions were formed by negative chemical ionisation (nCI) with methane as mediator. For the accumulation (typical accumulation time 5 ms) of fluoranthene reactant anions in the ion trap, the polarity was switched to negative mode. Glycopeptide cations and fluoranthene anions were incubated in the ion trap for 70 ms, allowing electron transfer, followed by the registration of the ETD fragment ion spectrum for *m/z* 150 to 3000. Selected MS/MS spectra of CID and ETD were interpreted manually using Bruker Daltonics Data Analysis software (Bruker Daltonics).

### Protein sequence alignment

Multiple sequence alignment of selected proteins was performed using Clustal Omega [50] using default parameters. Pairwise sequence alignment was carried out using EMBOSS matcher, based on the Bill Pearson's align application, version 2.0u4 [51].

### Accession numbers

BN1106_s6570B000051

BN1106_s4187B000061

Fh_Contig2249

BN1106_s1922B000122

BN1106_s7612B000030

BN1106_s1518B000071

BN1106_s7307B000022

BN1106_s25B000189

BN1106_s1612B000138

BN1106_s462B000766

BN1106_s2763B000063

BN1106_s3008B000074

BN1106_s1081B000242

BN1106_s5172B000090

BN1106_s666B000200

BN1106_s9461B000006

BN1106_s4565B000032

BN1106_s10139B000014

BN1106_s3227B000227

BN1106_s10667B000018

BN1106_s5100B000033

BN1106_s8462B000006

BN1106_s6570B000050

BN1106_s4482B000044

gi|27526823

## Results

### Mannose, fucose and the mucin-type core-1 (also known as T-antigen) were the main terminal carbohydrate motifs detected and localised by lectin fluorescence and lectin blot techniques

**Fig 1:**
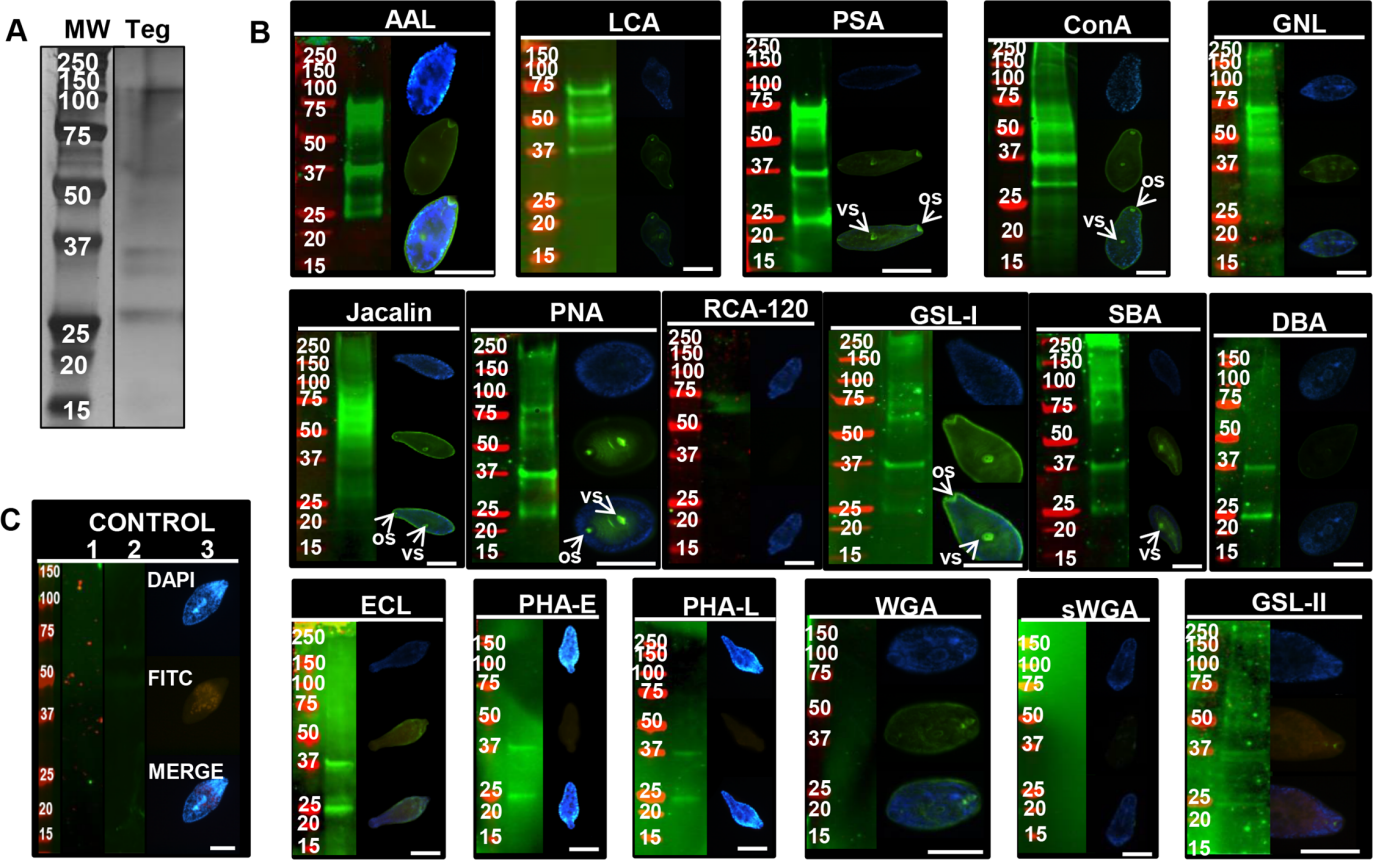
Glycoprotein detection and localisation on *F*. *hepatica* NEJTeg by lectin blot and lectin fluorescence staining. The total protein profile of *F*. *hepatica* NEJTeg (Teg) was revealed after SDS-PAGE fractionation and visualised in-gel by silver staining (**A**). Fixed NEJ and nitrocellulose membranes were incubated with FITC-labelled or biotinylated-conjugate lectins respectively. Specific lectin binding to glycans with terminal Fuc- (AAL, LCA, PSA), Man- (ConA, GNL), Galβ1-3GalNAc- (Jacalin, PNA), Gal/GalNAc- (RCA-120, GSL-I, SBA, DBA), Lac- (ECL), oligosaccharides- (PHA-E, PHA-L) or GlcNAc- (WGA, sWGA, GSL-II) motifs is shown. Oral sucker (or) and ventral sucker (vs) are identified with arrows (**B**). Negative controls consisted of nitrocellulose membranes without lectin incubation (1), nitrocellulose membranes incubated with biotinylated-conjugate lectins that were previously incubated with their specific inhibitors (2) and fixed NEJ incubated with FITC-labelled lectins that were previously incubated with their specific inhibitors (3). The list of lectins used and their corresponding inhibitors are detailed in Table 1 (**C**). Nitrocellulose membranes were exposed to IRDye-labelled streptavidin to reveal positive lectin binding (green) at various molecular weights (markers in red). Glycoprotein profiles were visualised by IR system. Epifluorescence microscope was employed to detect glycan localisation (green) and DNA was counterstained with DAPI (blue). Merged image of both micrographs is included for each lectin. Scale bar = 100 μm

The main components of the *F*. *hepatica* Teg are glycoproteins [52]. However, the nature of the different glycans and the identity of individual glycoproteins are still unknown. For that reason, a panel of 17 fluorescein-labelled plant lectins were employed in fluorescence microscopy experiments for glycan localisation (Fig 1). In parallel, lectin blots were used to identify and compare protein bands carrying glycans.

The most dominant protein bands detected in NEJTeg by silver staining had an apparent molecular weight between 25–37, 50–75 and 100 kDa (Fig 1A). This was consistent with the broad range of bands detected in the blots (Fig 1B), indicating that the majority of the NEJTeg components were glycoproteins. Indeed, this recognition was probed with the mannose (Man)-binding lectins ConA and GNL, all Man and fucose (Fuc)-binding lectins (AAL, LCA and PSA), the Gal- binding lectins GSL-I and all the T-antigen-binding lectins (PNA and Jacalin) suggesting the abundance of these terminal carbohydrate motifs on these NEJTeg-derived glycoproteins. The exclusive 25 and 37 kDa pattern detected by the lectins PHA-L, PHA-E, GSL-II, SJA, DBA, ECL (Fig 1B), UEA and SJA incubated membranes (S1 Fig), suggested the presence of complex glycan mixture and complex glycosylation patterns in these protein bands.

Lectin binding revealed that glycans were distributed uniformly on the parasite surface, but most intensely on the tegumental spines. Strong carbohydrate recognition at the oral and ventral suckers was detected by PNA, Jacalin, ConA, PSA and GSL-I (Fig 1B), suggesting the possible role of Man, Fuc and Gal/GalNAc decorated glycoproteins in adhesion and nutrient intake from the host. Another lectin (SBA) bound to a variety of high molecular sized glycoproteins (Fig 1B) with terminal Gal or GalNAc located at the ventral sucker and its periphery.

### GSL-I, SBA, PNA and Jacalin lectins exhibited differences in glycosylation between NEJTeg and NEJSom

**Fig 2:**
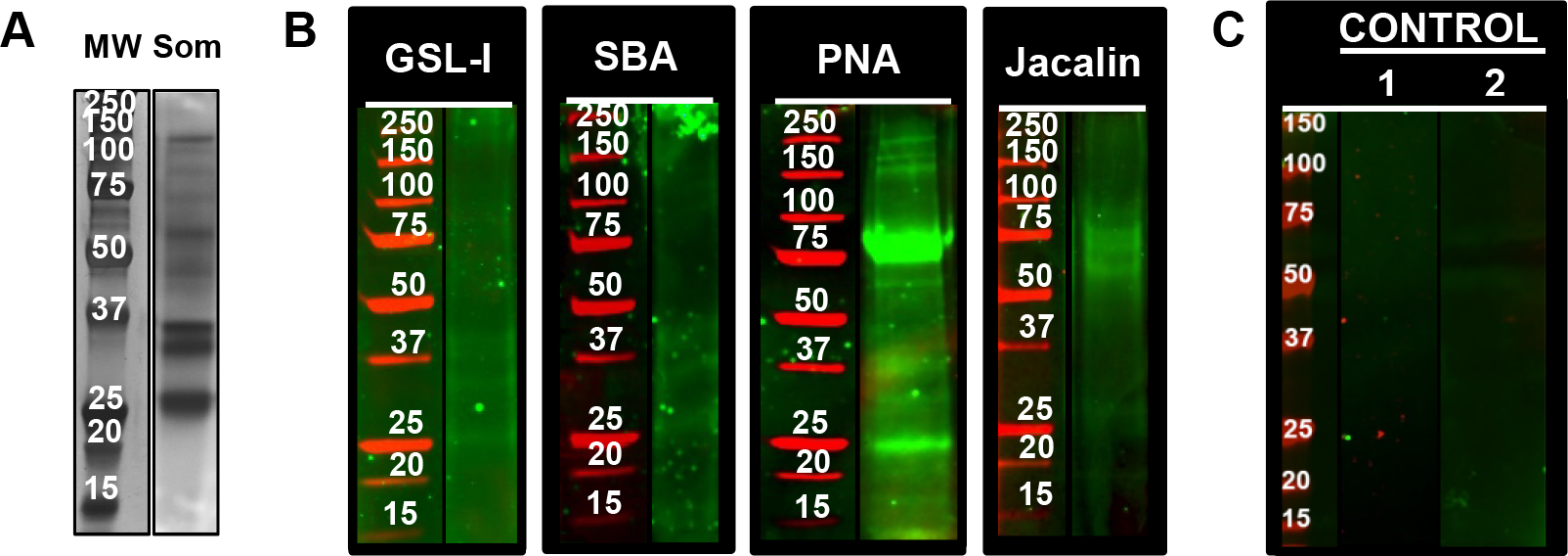
Glycosylation of *F*. *hepatica* NEJSom. The total protein profile of *F*. *hepatica* NEJSom (Som) was revealed after SDS-PAGE fractionation and visualised in-gel by silver staining **(A).** NEJSom preparations were SDS-PAGE fractioned, transferred to nitrocellulose membranes and incubated with the biotinylated-labelled lectins GSL-I, SBA, PNA and Jacalin (**B**). Negative controls consisted of nitrocellulose membranes without lectin incubation (1) and nitrocellulose membranes incubated with biotinylated-conjugate lectins that were previously incubated with their specific inhibitors (2). The list of lectins used and their corresponding inhibitors are detailed in Table 1 (**C**). An additional incubation with IRDye-labelled streptavidin was used to detect positive lectin binding at different molecular weights (red markers). Glycoproteins were revealed by infrared imaging.

To provide additional information regarding the distribution of *F*. *hepatica* glycoproteins, NEJTeg and NEJSom fractions were compared by silver staining and lectin blots. Similarities observed by silver staining in the migratory components between the NEJTeg (Fig 1A) and NEJSom (Fig 2A) and by eleven lectin blots indicated that there were some (glyco)proteins shared in both extracts. In addition, a small group of lectins did not recognise glycoproteins in any of the NEJ extracts, i.e. the Gal-binding lectin RCA-120, all NeuAc-binding lectins (SNA and MAL-II) and the majority of the GlcNAc-binding lectins (DSL, LEL and STL) (S1 Fig), Nevertheless, the lectins GSL-I, SBA, PNA and Jacalin revealed significant differences in glycosylation of NEJSom (Fig 2B). For example, in the NEJSom, PNA, which shows a similar pattern of recognition as Jacalin, bound the 37 kDa band as observed in the NEJTeg, but also two additional prominent (glyco)protein bands at 25 and 75 kDa. These results suggest the presence of T-antigen in the internal organs of the NEJ.

### High mannose and oligomannose are the most dominant *N-*glycans in NEJTeg

**Fig 3:**
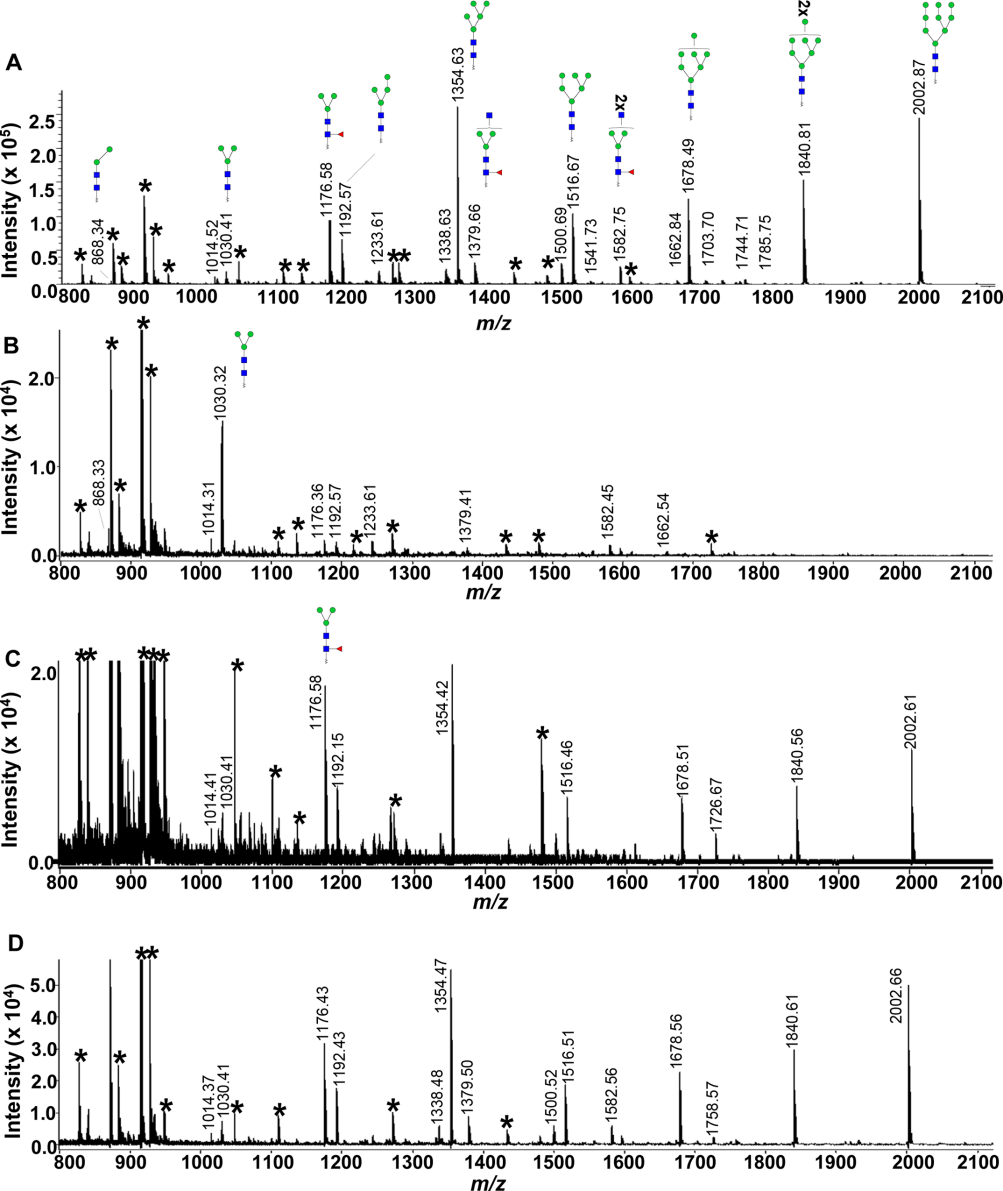
MALDI-TOF-MS spectra of AA-labelled *N-*linked glycans of *F*. *hepatica* NEJTeg before and after exoglycosidase treatments. N-glycans released from NEJTeg using PNG-F were labelled with fluorophore 2-aminobenzoic acid (2-AA). MS spectra of AA-labelled *N-*glycans were acquired by MALDI-TOF-MS without exoglycosidase treatment (**A**) and after α-mannosidase (**B**), β-*N*-acetylglucosaminidase (**C**) and β-galactosidase (**D**) treatments. Unidentified peaks are represented with an asterisk (*). Green circle, Man; blue square, GlcNAc; red triangle, Fuc.

As the NEJTeg showed high (glyco)protein abundance and complexity, we decided to perform an in-depth characterisation of the protein-derived glycans of this preparation by MALDI-TOF-MS. In order to characterise the tegumental *N-*glycans, these were enzymatically released and MALDI-TOF-MS spectra were recorded before and after specific exoglycosidase treatments (Fig 3).

The spectrum obtained from PNG-F released *N-*glycans showed 5 dominant peaks with *m/z* values 1354.63, 1516.69, 1678.49, 1840.81 and 2002.87 [M−H]^−^ (Fig 3A). These peaks corresponded to glycans with composition H5N2, H6N2, H7N2, H8N2 and H9N2 (hexose (H), N-acetylhexosamine (N)) respectively, suggesting, in line with the previous lectin binding affinity results, a clear dominance of high mannose *N-*glycans in NEJTeg. This composition was confirmed after exoglycosidase treatment as all these dominant peaks completely disappeared or drastically decreased after α-mannosidase treatment while peak *m/z* 1030.32 [M−H]^−^, which corresponded to the *N-*glycan with composition H3N2, became the most dominant ones in the spectrum (Fig 3B).

**Fig 4:**
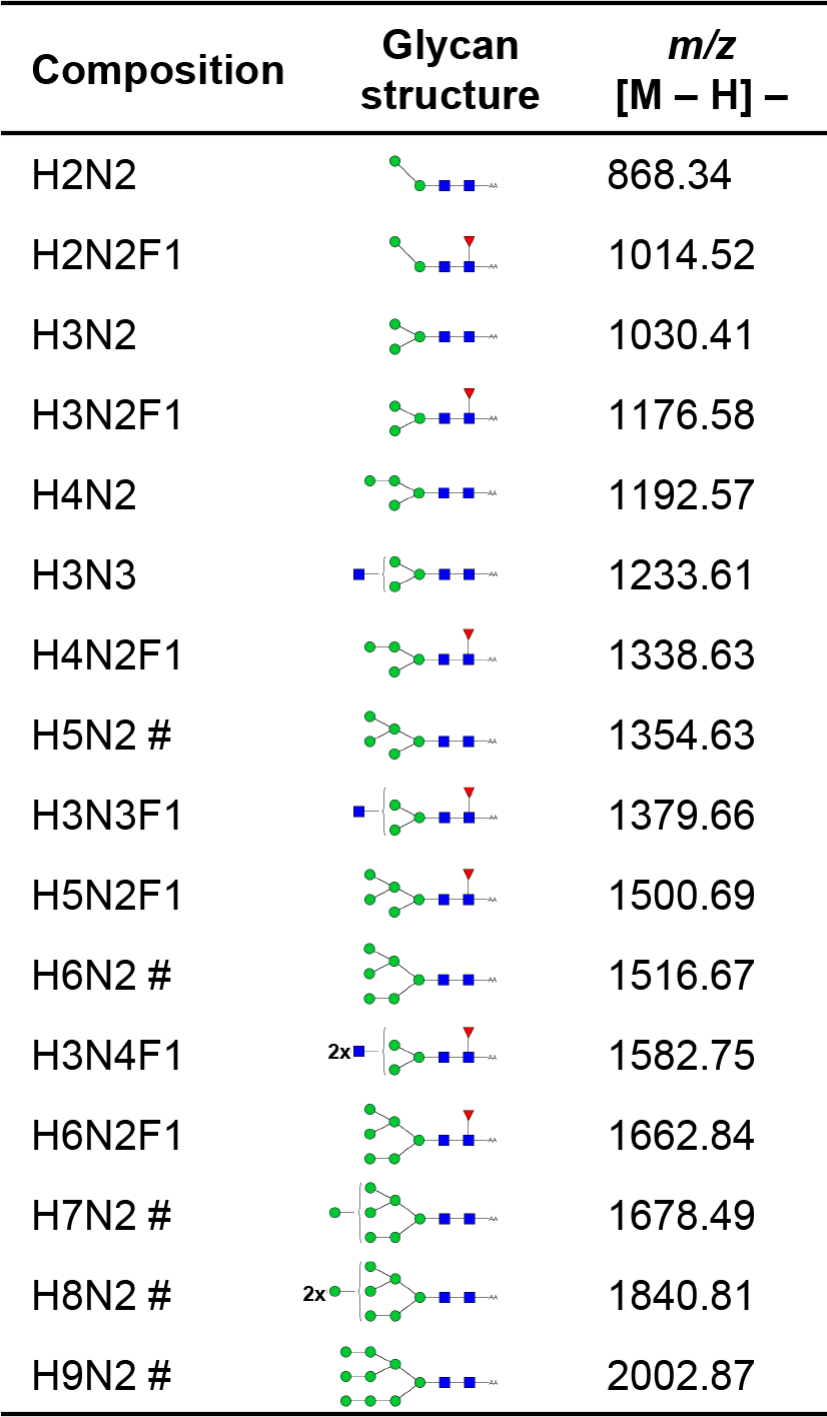
List of the characterised *F*. *hepatica* NEJTeg *N-*glycans identified by MALDI-TOF-MS. Composition of glycan subsets expressed in terms of hexose (H), *N-*acetylhexosamine (N) and deoxyhexose (F). The five most prominent peaks shown in Fig 3A are indicated (**#**). Red triangle, Fuc; green circle, Man; yellow circle, Gal; blue square, GlcNAc; yellow square, GalNAc.

Some compositions suggestive of complex type *N-*glycans were detected in the MALDI-TOF MS of the NEJTeg preparation, with lower relative abundance. The comparison of the PNG-F spectra before and after β-*N-*acetylglucosaminidase treatment confirmed the presence of terminal, unsubstituted GlcNAc on the antennae of glycans with *m/z* 1379.66 [M−H]^−^ (H3N3F1) (deoxyhexose (F)) and *m/z* 1582.75 [M−H]^−^ (H3N4F1) seen in Fig 3A but undetectable in Fig 3C. This observation also confirms that the Fuc residue found in these glycans is attached to the chitobiose core. We assume that the Fuc is α1-6-linked to GlcNAc-1 since α1-3-linkage would have rendered the glycans resistant to PNG-F. Both PNG-F glycan spectra before and after β-galactosidase treatment were very similar suggesting that no terminal β-Gal was present which was in line with the negative binding detection of RCA-120 lectin. The spectrum obtained from PNG-A released *N-*glycans (S2 Fig) showed a similar glycan pattern as the PNG-F release indicating that no specific PNG-F resistant core α1–3 fucosylated glycans are present in NEJTeg. All peaks identified and their corresponding glycan structures are described in Fig 4.

### The NEJ FhCL3 and FhCB3 were detected in the glycosylated NEJTeg fraction alongside other important immunomodulatory molecules

To specifically identify which NEJTeg proteins are glycosylated, we biotinylated the glycan structures contained in NEJTeg glycoproteins and then selectively isolated the glycoprotein components by affinity monomeric avidin chromatography from the total NEJTeg protein pool. The efficiency of the glycoprotein biotin-labelling (B-NEJTeg) and isolation of the proteins (A-UB-NEJTeg) and glycoproteins (A-BB-NEJTeg) was confirmed by comparing the protein profile between fractions using silver staining and the streptavidin blot (S3 Fig).

Proteomic MS analysis identified a total of 20 proteins in B-NEJTeg and A-BB-NEJTeg preparations whose molecular weights correlated with protein bands found by silver staining. A total of eighteen proteins were identified in the A-BB-NEJTeg (glycosylated) fraction (Table 2). There were potentially important *F*. *hepatica* molecules identified in this fraction including the asparaginyl endopeptidase legumain-1, the antioxidants GST and PRX and heat shock protein 70 (HSP70). It is noteworthy also to highlight the identification of the proteases FhCB3 and FhCL3 in the glycosylated fraction. Two isoforms of FhCL3 were identified in this extract. All the glycoproteins except GST and Histone H4 were predicted to contain potential *N-* glycosylation sites. GST and Histone H4 were predicted not to contain *N-*glycosylated sites, suggesting that they are *O-*glycosylated.

**Table 2:** Characterisation of *F*. *hepatica* A-BB-NEJTeg glycoprotein composition detected following glycoprotein biotinylation, affinity chromatography isolation and LC-MS/MS analysis.

Protein	Identifier	ID prob (%)	UP B-NEJTEG	UP A-BB-NEJTEG	Pot. *N-*Glyc
FhCB3	BN1106_s6570B000051	100	5	8	4
FhCL3	BN1106_s4187B000061	100	4		0
PRX	Fh_Contig2249	100	2	3	4
Paramyosin	BN1106_s1922B000122	100	2		3
Legumain-1	BN1106_s7612B000030	100		9	1
Fructose-bisphosphate aldolase	BN1106_s1518B000071	100		5	1
Uncharacterised protein	BN1106_s7307B000022	100		5	14
Basement membrane-specific heparan sulfate proteoglycan core protein	BN1106_s25B000189	100		3	27
Multi-domain cystatin	BN1106_s1612B000138	100		3	10
Cell polarity protein; Neurexin-1-α; Fibropellin-1	BN1106_s462B000766	100		3	2
Protein disulphide isomerase	BN1106_s2763B000063	100		3	1
FhCL3	BN1106_s3008B000074	100		3	1
GST-Sigma	BN1106_s1081B000242	100		3	0
Uncharacterised protein	BN1106_s5172B000090	100		2	8
Lysosomal α-mannosidase	BN1106_s666B000200	100		2	8
HSP70	BN1106_s9461B000006	100		2	5
Estrogen-regulated protein EP45	BN1106_s4565B000032	100		2	2
FhCL3	BN1106_s10139B000014	100		2	1
Enolase	BN1106_s3227B000227	100		2	1
Histone H4	BN1106_s10667B000018	100		2	0

Potential protein *N-*glycosylation was determined by assessing the presence of *N-*glycan consensus sequons by the NetNGlyc. server

ID prob (identity probability), UP (unique peptide), B-NEJTEG (biotinylated NEJ tegument), A-BB-NEJTeg (avidin bound biotinylated NEJ tegument), Pot *N-*Glyc (number of potential *N-*glycosylation sites)

### Most of the NEJ specific cathepsins have been shown to contain potential *N-*glycosylation

Cathepsins were the most abundant proteins found in the glycosylated fraction; due to their pivotal role in parasite survival and since cathepsins from the same family have been employed as vaccine candidates, we investigated the total NEJTeg material focusing on evaluating cathepsin-specific glycosylation at the glycopeptide level.

**Fig 5:**
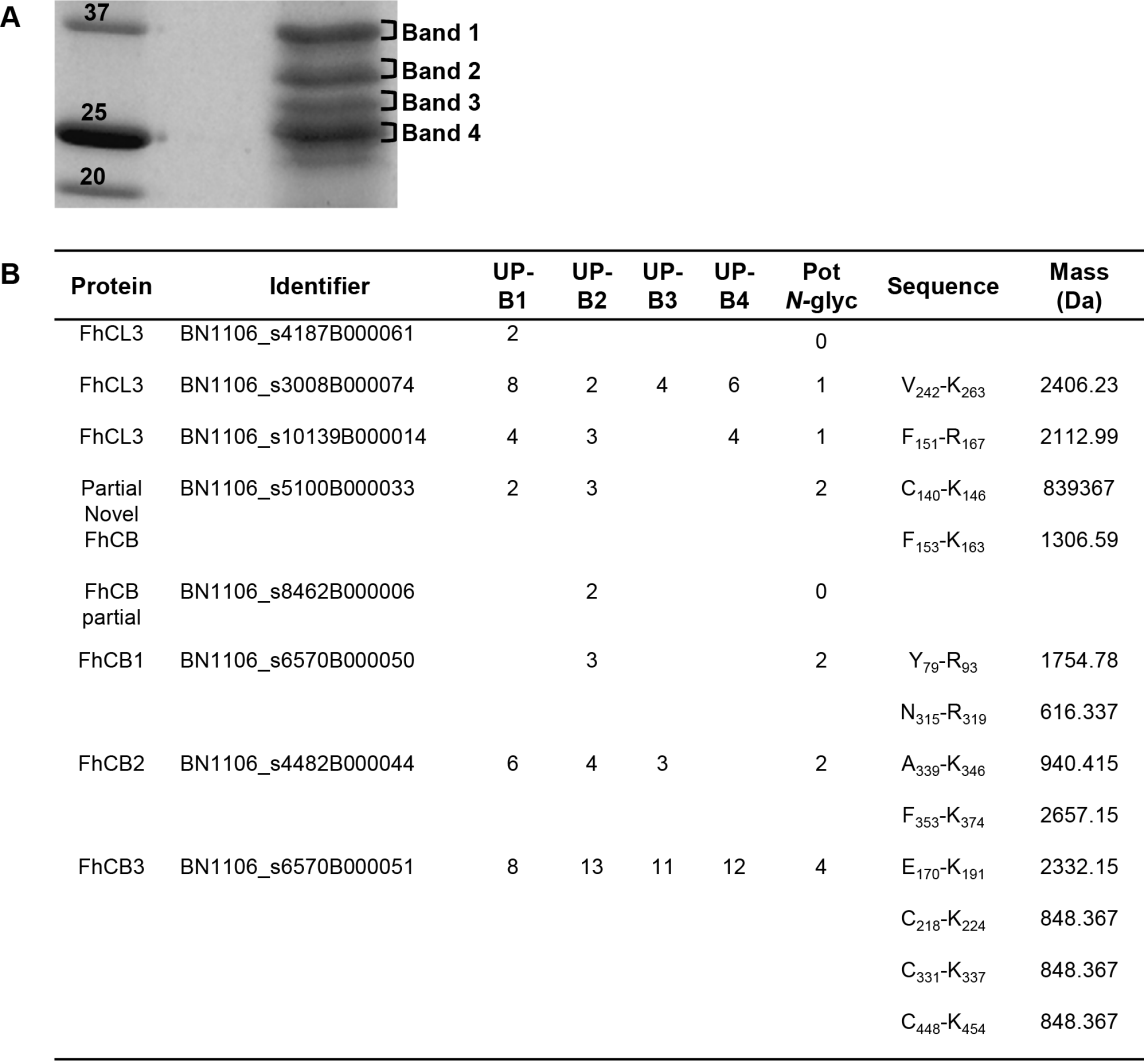
Identification of *F*. *hepatica* cathepsins in NEJTeg. NEJTeg was loaded in SDS-PAGE and stained with Coomassie Blue in order to stain protein bands (**A**). Bands 1 (B1), 2 (B2), 3 (B3) and 4 (B4), were excised, tryptic digested and identified with LC-MS/MS analysis. Potential *N-*glycosylation sites (Pot *N-*gly) and monoisotopic mass of the tryptic peptides containing glycosylation were predicted (**B**). UP (unique peptide).

After selecting and excising bands found at the predicted molecular weight of the cathepsins (Fig 5A), tryptic in-gel digestion and protein identification was performed in order to select cathepsins to be examined in detail. We were able to identify several cathepsins, including FhCL3, FhCB1, FhCB2, FhCB3 and partial novel FhCB protein (Fig 5B). The NetNGlyc 1.0 server suggested the presence of one potential *N-*glycosylation site in the amino acid sequence of two of the FhCL3 members (BN1106_s3008B000074 and BN1106_s10139B000014), at the positions N151 and N255, respectively. FhCB1, FhCB2 and the partial novel FhCB were predicted to contain two *N-*glycosylation sites at positions N145 and N156 for partial novel FhCB, N80 and N315 for FhCB1 and N345 and N356 for FhCB2. FhCB3 was predicted to have four *N-*glycosylation sites at positions N180, N223, N336 and N453. However, one of the FhCL3 (BN1106_s4187B000061) and the partial FhCB (BN1106_s8462B000006) were predicted not to be *N-*glycosylated. When 0 missed cleavages were allowed, tryptic peptide masses containing potential *N-*glycosylation vary from 617.337 to 2658.15 kDa.

Once the cathepsin members and the relevant tryptic peptides with potential *N-*glycosylation sites were identified, we wanted to determine whether these glycosylation sites were occupied by glycans and also the composition of the glycans involved.

### One of the glycosylation sites of the FhCB1 and one of the FhCL3 proteins is occupied by a Manα1-3/6Manα1-4GlcNAcβ1-4GlcNAcβ1- *N-*glycan

**Fig 6:**
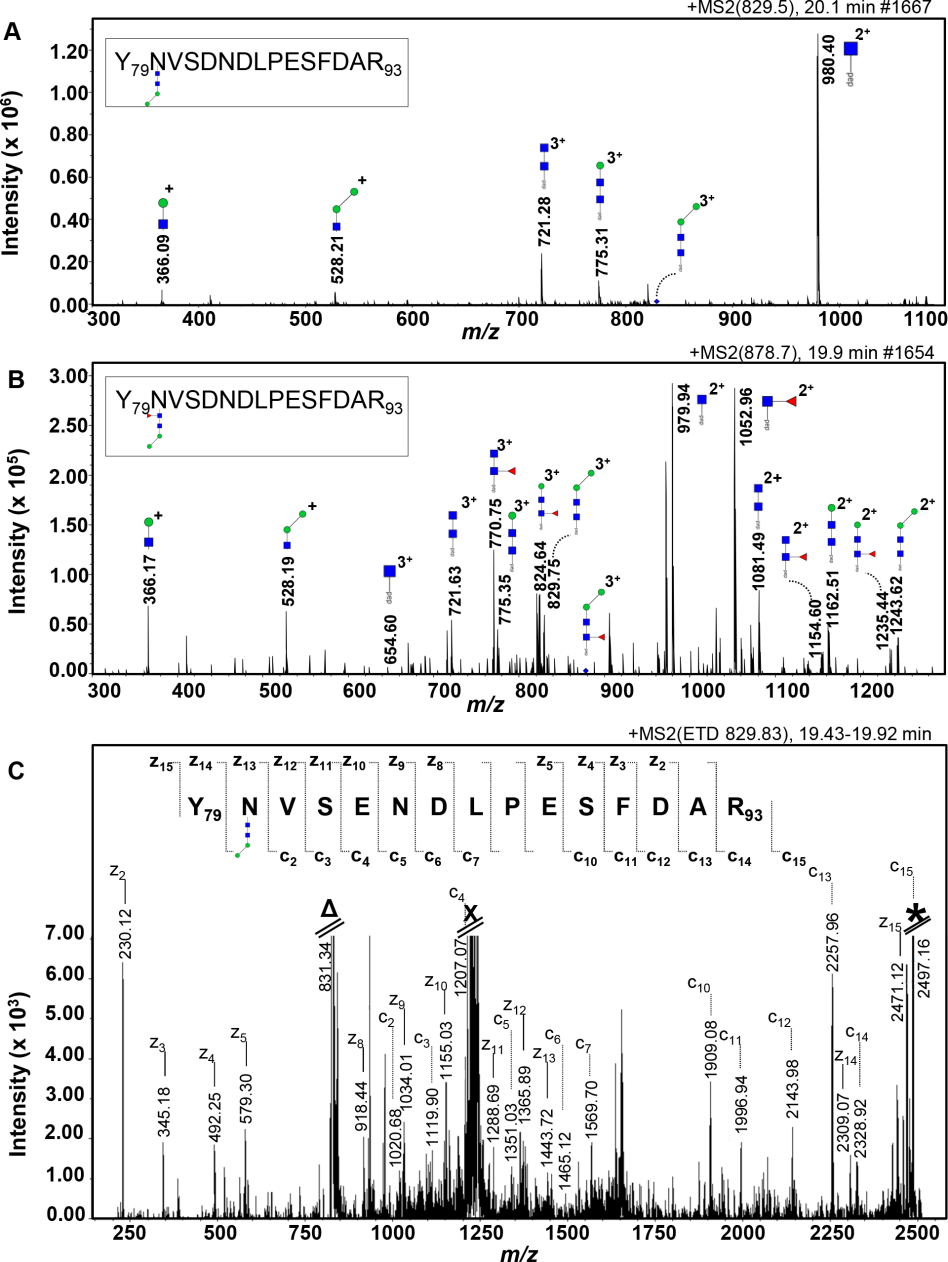
Identification of short *N-*glycans occupying an *N-*glycosylation site of FhCB1. Nano-RP-LC-ESI-ion trap-MS/MS with collision-induced dissociation (**A** and **B**) and with electron transfer dissociation (**C**) of the tryptic glycopeptide Y_79_NVSENDLPESFDAR_93_ from FhCB1 (BN1106_s6570B000050). The [M+3H]^3+^ parent ions (blue diamonds) at *m/z* 829.50 (**A**) and 878.70 (**B**) of the glycopeptide carrying a glycan of composition H2N2 and F1H2N2 respectively were selected. Fragment ions are indicated. The residual signals at the *m/z* corresponding to [H+3H]^3+^ (**Δ**), to the doubly charged ions that result from capture of 1 electron without dissociation [H+3H]^2+·^ (**X**) and to the singly charged ions that result from capture of 2 electrons without dissociation [H+3H]^+··^ (*) are indicated (**C**). Monoisotopic masses are given. Man (green circle), GlcNAc (blue square) and Fuc (red triangle), pep (peptide moiety).

All tryptic glycopeptides from the four bands were applied to a RP-nano-LC column coupled to an ion-trap MS/MS system and fragmented in the auto-MS/MS mode. A parent-ion of *m/z* 829.50 [M+3H]^3+^ in the 20.1 min elution was detected in band 2, giving rise to oxonium glycan fragment ions at *m/z* 366.09 [M+H]^+^ (H1N1) and *m/z* 528.21 [M+H]^+^ (H2N1), which indicated the presence of the terminal di-mannosylated HexNAc element. Further inspection of the CID-MS/MS spectrum of the parent ion *m/z* 829.50 [M+3H]^3+^ indicated the presence of a glycan with the composition H2N2 linked to a peptide of 1755 Da (Fig 6A). [M+3H]^3+^ fragment ions were observed for this peptide carrying N2 (*m/z* 721.28) or a H1N2 trisaccharide (*m/z* 775.31) structure of an Asn-linked glycan. Furthermore, a [M+2H]^2+^ fragment ion at *m/z* 980.40 (N1-peptide) was fully in line with the presence of an *N-*glycan.

The CID-MS/MS spectrum of another parent ion *m/z* 878.70 [M+3H]^3+^ in the 19.9 min elution indicated the presence of another sort of glycan with the composition F1H2N2 linked to a peptide with the same mass as the previous peptide described (Fig 6B). [M+3H]^3+^ fragment ions were observed for this peptide carrying a Fuc attached to the N2 (*m/z* 770.75) or a F1H1N2 tetrasaccharide (*m/z* 824.64) structure of an Asn-linked glycan. In addition, a series of [M+2H]^2+^ fragment ions at *m/z* 1243.62 (H2N2-peptide), 1235.44 (F1H1N2-peptide), 1162.51 (H1N2-peptide), 1154.60 (F1N2-peptide), 1081.49 (N2-peptide), 1052.96 (F1N1-peptide) and 979.94 (N1-peptide) were fully in line with the presence of the fucosylated version of the dimannosyl core *N-*glycan.

The mass of the peptide containing the N2H2 and F1N2H2 *N-*glycans matched the mass of one of the tryptic peptides of FhCB1 (BN1106_s6570B000050). To obtain information on the glycopeptide sequence and identify the cathepsin that contained the *N-*glycan, the ETD-MS/MS spectra of the same parent ions *m/z* 829.83 [M+3H]^3+^ and 878.7 [M+3H]^3+^ were recorded in which peptide cleavages were predominantly observed while leaving the glycosidic linkages intact [53]. For the parent ion *m/z* 829.83 [M+3H]^3+^ (Fig 6C), the c’-type as well as z’-type ions arising from peptide bond cleavages provide partial sequences of the peptide backbone from the *N-* and *C-*terminal side, respectively. As annotated in Fig 6C, the VSENDLPESFDAR sequence can be read directly from the c_2_-c_15_ ion series, while the signals indicated with z_2_-z_15_ read the DFSEPLDNESVN(glycan)Y sequence, including the mass increment of 732 Da accountable to the glycan H2N2_._ This sequence is the FhCB1 tryptic peptide (no missed cleavage) Y_79_NVSENDLPESFDAR_93_, containing the *N-*glycosylation consensus sequence NVS. On the other hand, when the ETD-MS/MS spectrum of the parent ion *m/z* 878.7 [M+3H]^3+^ was recorded, the VSENDLPESFDAR sequence containing the glycan F1N2H2 could not be directly read. Taken together, the MS/MS data indicated that FhCB1 is modified at the Asn in position N80 with a Manα1-3/6Manα1-4GlcNAcβ1-4GlcNAcβ1-Asn *N-*glycan. Nevertheless, despite the strong evidence of the presence of a monofucosylated version of the same *N-*glycan attached to the FhCB1 in CID-MS analysis, its confirmation remained inconclusive by ETD-MS.

**Fig 7:**
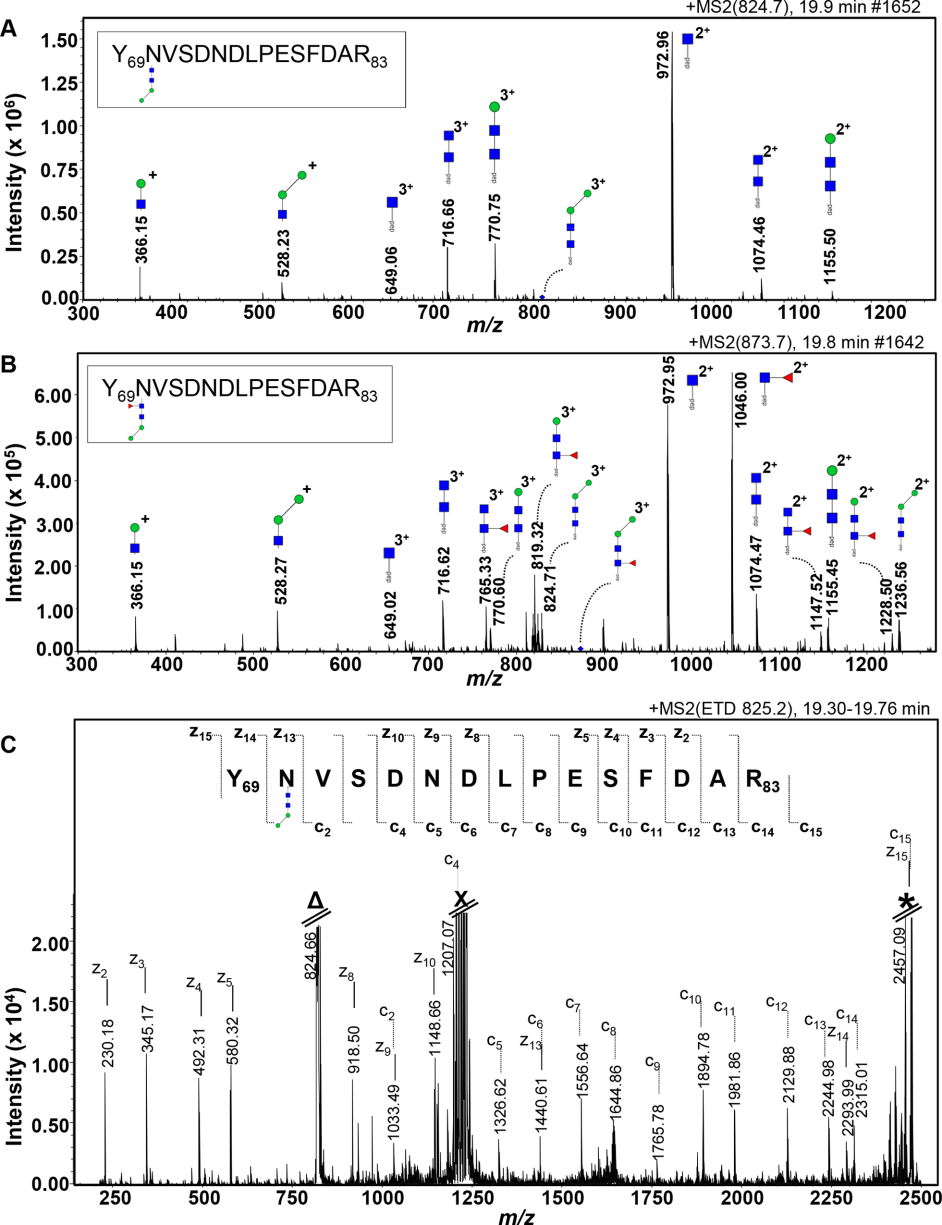
Identification of short *N-*glycans occupying an *N-*glycosylation site of on the YNVSENDLPESFDAR tryptic peptide. Nano-RP-LC-ESI-ion trap-MS/MS with collision-induced dissociation (**A** and **B**) and with electron transfer dissociation (**C**) of the tryptic glycopeptide YNVSENDLPESFDAR from FhProCB2 (gi|27526823). The [M+3H]^3+^ parent ions (blue diamonds) at *m/z* 824.70 (**A**) and 873.70 (**B**) of the glycopeptide carrying a glycan of composition H2N2 and F1H2N2 respectively were selected. Fragment ions are indicated. The residual signals at the *m/z* corresponding to [H+3H]^3+^ (**Δ**), to the doubly charged ions that result from capture of 1 electron without dissociation [H+3H]^2+·^ (**X**) and to the singly charged ions that result from capture of 2 electrons without dissociation [H+3H]^+··^ (*) are indicated (**C**). Monoisotopic masses are given. Man (green circle), GlcNAc (blue square) and Fuc (red triangle).pep, peptide moiety.

For band 2, the presence of the H2N2 and F1H2N2 *N-*glycans attached to a peptide of 1741.2 kDa were detected in the chromatograms of the parent-ions of *m/z* 824.70 [M+3H]^3+^ in the 19.9 min elution and *m/z* 873.70 [M+3H]^3+^ in the 19.8 min elution respectively (Fig 7A and 7B). The annotation of the ETD-MS/MS spectrum of the same parent ion *m/z* 825.20 [M+3H]^3+^ showed the VSDNDLPESFDAR sequence from the c_2_-c_15_ ion series and the DFSEPLDNDSVN(glycan)Y sequence from the z_2_-z_15_ ion series, including the mass increment of 732 Da of the glycan H2N2 (Fig 7C). This sequence is slightly different to the sequence of the tryptic peptide identified for FhCB1 (no missed cleavage) as there is a replacement of the glutamic acid (E) in position 83 by an aspartic acid (D). As the new sequence was not identified in the list of tryptic peptide containing *N-*glycosylation, the sequence tag YNVSDNDLPESFDAR was analysed by BLAST (NCBI nr database). The result showed a 100% coverage and identity hit with FhProCB2 (gi|27526823) which contains the *N-*glycosylation consensus sequence NVS in position N70. Following identification of further cathepsin B class proteins for *F*. *hepatica* and the comparative analysis of those cathepsin sequences publically available, the FhProCB2 (gi|27526823) is consistent with the FhCB1 sequence (S1 File, S1 Table). The slight difference (represented as at most 2 SNPs resulting in the amino acid change from aspartic acid to glutamic acid) between the tryptic peptide YNVSDNDLPESFDAR and the sequence corresponding to FhCB1 sequence in the *F*. *hepatica* genome and that submitted to Genbank is most likely due to isolate differences between the various fluke samples used for these studies.

As demonstrated for FhCB1, FhCL3 also showed firm evidence of the same glycosylation pattern. The fragmented ions confirming the glycan structures are described in Table 3. As with the analysis performed in tryptic peptides found in band 2, tryptic glycopeptides of the FhCL3 (BN1106_s10139B000014, scaffold10139) were detected in bands 1 and 4. The same glycan structures seen for FhCB1 were found to be linked to a peptide of 2113.9 kDa in the chromatograms of the parent-ions of *m/z* 949.20 and 997.80 [M+3H]^3+^ in the 22.2 and 22.1 min elution respectively. To investigate this further for the tryptic peptide derived from FhCL3, ETD-MS/MS for the same parent ion in the same band was carried out. The results showed that the ion c_2_-c_17_ and z_4_-z_17_ ion series have correspond to the sequences N(glycan)QTLFSEQQVLDCTR and VLQQESFLTQN(glycan)QF respectively, taking into consideration the mass increment of the H2N2 glycan. This sequence is the FhCL3 tryptic peptide (no missed cleavage) F_151_QNQTLFSEQQVLDCTR_167_, containing the *N-*glycosylation consensus sequence NQT. No definitive identification of its fucosylated version could be achieved in the ETD-MS/MS analysis.

**Table 3:** List of the fragment ions after CID-MS/MS of parent ions of tryptic peptide of FhCL3 (BN1106_s10139B000014).

Parent ion *m/z* [M+3H]^3+^	Fragment ions *m/z*	Fragment charge state	Ion structure	Peptide Mass (Da)
**949.20**			H2N2-pept	2112.9
	773.13	[M+3H]^3+^	N1-pept	
	840.84	[M+3H]^3+^	N2-pept	
	894.81	[M+3H]^3+^	H1N2-pept	
	1159.31	[M+2H]^2+^	N1-pept	
	1260.61	[M+2H]^2+^	N2-pept	
**997.80**			F1H2N2-pept	2112.9
	840.77	[M+3H]^3+^	N2-pept	
	889.11	[M+3H]^3+^	F1N2-pept	
	894.72	[M+3H]^3+^	H1N2-pept	
	943.09	[M+3H]^3+^	F1H1N2-pept	
	948.75	[M+3H]^3+^	H2N2-pept	
	1159.14	[M+2H]^2+^	N1-pept	
	1232.16	[M+2H]^2+^	F1N1-pept	
	1260.61	[M+2H]^2+^	N2-pept	
	1333.68	[M+2H]^2+^	F1N2-pept	
	1341.57	[M+2H]^2+^	H1N2-pept	
	1414.68	[M+2H]^2+^	F1H1N2-pept	
	1422.75	[M+2H]^2+^	H2N2-pept	
	1496.77	[M+2H]^2+^	F1H2N2-pept	

Sixty four additional *N-*glycopeptide species were found in the CID-MS/MS spectra whose parent ions were registered in the elution times from 2.5 to 29.3 min (S2 Table). The glycans attached to those peptides matched those observed in the NEJTeg *N-*glycan profile (Fig 4). Nevertheless, the peptide mass to which the glycans were linked did not match with the masses of the remaining tryptic peptides derived from the cathepsins.

## Discussion

To date, this is the first study characterising the glycans attached to the proteins in the *F*. *hepatica* NEJTeg and NEJSom preparations by the systematic use of an extensive panel of lectins. In addition, a mass spectrometric glycan characterisation of the tegumental extract was performed.

The lack of sialic acid and the wide presence and distribution of high mannose *N-*glycans and α1–6 Fuc attached to the chitobiose core of oligomannose and truncated *N-*glycans in the NEJTeg was firstly detected by the binding patterns of the lectins ConA, GNL, PSA and LCA, and confirmed later on by MALDI-TOF MS which represent an important percentage of the total of NEJ *N-*glycans. The wide distribution of Man-rich *N-*glycans and the presence of fucosylated glycans among the NEJ glycoproteins have been found in other helminths including *O*. *viverrini* [18] *Schistosoma* spp. [54,55], *Haemonchus contortus* [56,57] and *Taenia solium* [58]. These glycans in *S*. *mansoni* are recognised and internalised by DC *via* the *C-*lectin type receptor (CLR) DC-specific ICAM-3-grabbing non-integrin (DC-SIGN) [59], suppressing DC maturation and therefore decreasing the secretion of pro-inflammatory cytokines. We propose that glycoconjugates with terminal Man-*N-*glycans in the NEJTeg are likely to interact with DCs and macrophages *via* Man-specific CLRs at the early stages of infection, impairing the DC function and augmenting the M2a population in the peritoneal cavity, as seen with adult *F*. *hepatica* homogenate or ES fractions [23–25], contributing to the development of the biased Th2 that is elicited in the mammalian host [25,36].

In our study, lectin affinity techniques in some cases revealed different results than those using other glycomics techniques with respect to identification of terminal carbohydrates. For instance, β-*N-*acetylglucosaminidase and β-galactosidase treated *N-*glycans did not show evidence of terminal β-Gal- or GalNAc- on hybrid or complex glycans in the MS spectra but did show positive recognition of terminal Gal/GalNAc- residues on the parasite surface. This may reflect a low abundance and potential masking by high mannose *N-*glycans which are very abundant in NEJTeg. As seen in lectin blots, not many glycoproteins contain these residues. Alternatively, lectin fluorescence staining may also indicate the presence of glycolipids as described in adult *F*. *hepatica* [29,30], which would not have been detected in our MS spectra results, or the *O-*glycosylation pattern of NEJTeg. Similar PNA recognition has been described in adult *F*. *hepatica* [27,31] and in the nematode *O*. *viverrini* where 80% of the *O*-glycan profile is based on the T-antigen structure [18]. Tn antigen was also detected by the binding lectin VVL, which was also described in the basal membrane of the Teg and in the caeca of the adult fluke [27]. Although the same author also described the presence of sialyl-Tn antigen in the adult stage, we did not detect terminal sialic acid in the NEJTeg by MAL-II and SNA.

Taking into account the presence of these glycans in the oral and ventral suckers of the parasite, we suggest that NEJ-derived glycoproteins decorated with terminal Man, Fuc and Gal/GalNAc are in close contact with host mucins and intestinal enterocytes. Based on PNA positive recognition at the parasite surface and around the suckers in *F*. *hepatica*, *O-*glycan motifs may also play a role in intestinal epithelial invasion by this parasite, as has been shown also in studies of protozoal infections [60–63].

Differences in NEJTeg and NEJSom glycosylation were confirmed with four lectins. However, we also observed similar protein profiles in silver stained gels and in most of the lectin blots. It is possible that some tegumental material remained in the somatic preparation or *vice versa*. Due to the size of the NEJ and the relatively large number of parasites needed to obtain enough material, it is very difficult to achieve a complete separation of NEJTeg and NEJSom. It is known that some proteins are present in both *F*. *hepatica* Teg and Som. For example, the protein PRX as well as paramyosin are found in both Teg and Som of adult *F*. *hepatica* [64–66], although these studies also may be compromised by the difficulties encountered in achieving complete separation of the two fractions.

This is the first report that suggests that *F*. *hepatica* PRX and GST, which have well known antioxidant and immunomodulatory properties [67–70], are glycoproteins. Particularly for GST *F*. *hepatica*, it was not predicted to be potentially *N-*glycosylated, suggesting that it could be *O-*glycosylated. The facts that (1) the *F*. *hepatica* NEJTeg *O-*glycan profile could not be confirmed in our previous analysis and (2) the *O-*glycan consensus is not as specific as that for *N-*glycans limited our attempts to analyse potential *O-*glycosylation sites. It is reasonable to assume that mucin-type core-1 *O-*glycan would be the most likely to be attached to the GST due to the positive lectin binding of proteins at molecular weights of GST. Further studies will be required to assess whether *O-*glycan structures are present and involved in the detoxification and of immunoregulatory activities of these molecules.

To our knowledge, this is the first report describing the specific glycosylation of the *F*. *hepatica* NEJ-specific cathepsin cysteine proteases. These molecules, which play an important role in tissue degradation, in parasite penetration in the host gut and in parasite feeding [37,71] are glycosylated by Manα1-3/6Manα1-4GlcNAcβ1-4GlcNAcβ1- *N-*glycan. Although there is some evidence of the presence of Fuc on the chitobiose core, our analysis was inconclusive due to restrictions on material. However, the presence of this glycan is shown in the PNG-F released glycan pool of NEJTeg. The confirmation of *N-*glycosylation of FhCL3 in our study differs from previous findings [37,72]. Five genes were found in the *F*. *hepatica* genome encoding FhCL3 [44], meaning that the proteins evaluated by previous workers and in the present study could be different. Methodological differences may also be responsible. Previously, glycosylation of FhCL3 was assessed by detecting differences in protein migration in SDS-PAGE gel after enzyme deglycosylation [37,72]. Because of the size and the mass of the *N-*glycan detected, which is lower than 1 kDa, it is very difficult to measure differences in protein migration in SDS-PAGE gels before and after deglycosylation. Therefore, this is not an accurate approach to verify protein glycosylation when the glycans present are small. The use of MS for glycan analysis is more sensitive and suitable for these purposes. The absence of *N-*glycosylation sites in other trematode CL-like proteases [73], as well the presence of glycans in other various cathepsin proteinases including those from *Trypanosoma brucei* Cathepsin B [74] and human Cathepsin V (also known as cathepsin L2) [75] has been reported.

Crucially, GST, PRX and FhCL3 have been proposed and used as potential vaccine candidates. When these molecules were used as vaccines their maximum efficacy was observed when antigens were used in their native, rather than recombinant, versions [11,15]. As the putative glycans attached to GST and PRX have not been characterised we suggest that further in-depth molecular glycomic analyses are necessary in order to prove the existence of *N-* and/or *O-* glycosylation on these vaccine candidate molecules. There is only one report in the literature where glycosylation of FhCL3 was taken into account for measuring vaccine efficacy. This account involved rats vaccinated with recombinant FhCL3 expressed by *Saccharomyces cerevisiae* and recombinant baculovirus [76]. The formation of hyper high mannose-type *N-*glycans by yeasts [77] and mammalian-like post-translational modifications by baculovirus vector expression systems [78] differs significantly from the *N-*glycan structure identified for the native FhCL3 in the present work. This could have an effect on the final protein folding and/or the differences in the antigenic epitopes that could exist between the native protein and recombinant versions, explaining lack of efficacy of recombinant vaccine candidates. It is possible that the presence of this unusual *N-*glycan attached to the FhCL3 peptide backbone is required for proper processing and presentation. Correct glycosylation is an important issue for the production of recombinant antigens of *F*. *hepatica* and other helminths [79]. Further work in which alternative strategies for protein expression systems could be applied in order to express cathepsins with their correct glycosylation profiles and again, ascertain their importance for vaccine efficacy, is highly desirable.

In conclusion, we have demonstrated the presence of carbohydrates in the *F*. *hepatica* NEJTeg and NEJSom, with differences in glycosylation between the two fractions. We also have performed a glycan characterisation of the NEJTeg extract showing that high mannose and oligomannose *N-*glycans are the most dominant glycan structures. Finally, this work has shown that a set of important immunomodulatory proteins from *F*. *hepatica* NEJ, including some of the cathepsin family clade, are glycosylated. Knowledge of the specific structures of the glycans decorating these target proteins is particularly valuable for enhancing our understanding of immunoevasion and parasite migration as well as optimising the efficacy of future vaccines.

## Supporting Information

S1 Fig Glycoprotein detection of *F*. *hepatica* NEJTeg and NEJSom by lectin blot. Both NEJTeg (1) and NEJSom (2) preparations were SDS-PAGE fractioned, transferred to nitrocellulose membranes and incubated with the biotinylated-labelled lectins UEA, SJA, VVL, STL, LEL, DSL, SNA, MAL-II and RCA. An additional incubation with IRDye-labelled streptavidin was used to detect positive lectin binding at different molecular weights (MW). Glycoproteins were revealed by infrared imaging.
(TIF)

S2 Fig MALDI-TOF-MS spectra of AA-labelled *N-*linked glycans of *F*. *hepatica* NEJTeg released using PNG-A. N-glycans released from NEJTeg using PNG-A were labelled with fluorophore 2-aminobenzoic acid (2-AA). MS spectra of AA-labelled *N-*glycans were acquired by MALDI-TOF-MS. Unidentified peaks are represented with an asterisk (*).
(TIF)

S3 Fig Confirmation of NEJ glycoprotein biotinylation and isolation. NEJTeg (1) was employed for glycoprotein biotinylation. Biotinylated-NEJTeg (B-NEJTeg) (2) was used for biotin-avidin affinity chromatography, separating avidin-unbound-NEJTeg (A-UB-NEJTeg (3) and the avidin-bound-NEJTeg (A-BB-NEJTeg) (4) fractions. (A) SDS-PAGE and silver staining was used to detect the protein profiles of the four preparations. (B) FITC labelled-streptavidin-incubated membrane was used to confirm correct glycoprotein biotinylation of B-NEJTeg and correct biotin-avidin affinity chromatography separation.
(TIF)

S1 File Multiple sequence alignment of the *F*. *hepatica* NEJ cathepsins. Multiple sequence alignment of FhCB (BN1106_s5100B000033), FhCB1 (BN1106_s6570B000050), FhCB2 (BN1106_s4482B000044) and FhCB3 (BN1106_s6570B000051) and ProCB2 (gi|27526823) were performed using Clustal Omega. Sequences are ordered according to aligned. Differences in the region containing the *N-*glycosylation site are highlighted in yellow. Consensus *N-*glycosylation is underlined.
(PDF)

S1 Table Comparison of the protein amino acid sequence of the FhCBs to gi|27526823. (PDF)

S2 Table Annotation of the additional glycopeptides identified in NEJTeg at bands 1, 2, 3 and 4 that did not correspond to the cathepsins. (PDF)
