# Supplementary figures and images for: Tegument Glycoproteins and Cathepsins of Newly Excysted Juvenile Fasciola hepatica Carry Mannosidic and Paucimannosidic N-glycans
Source: PLoS Negl Trop Dis. 2016 May 3;10(5):e0004688. doi: 10.1371/journal.pntd.0004688 (PMC4854454; doi:10.1371/journal.pntd.0004688)

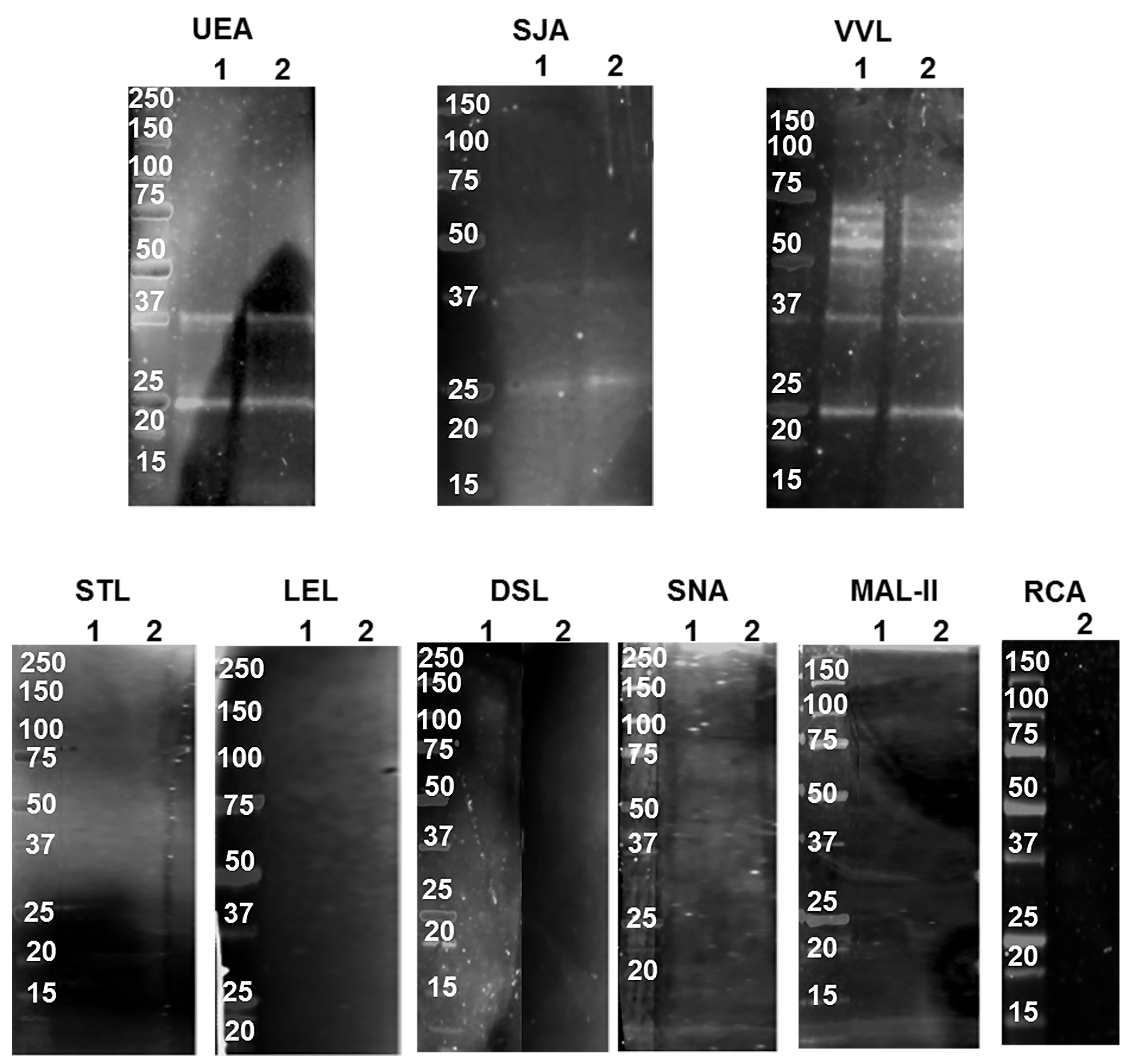

Supplement: S1 Fig — Both NEJTeg (1) and NEJSom (2) preparations were SDS-PAGE fractioned, transferred to nitrocellulose membranes and incubated with the biotinylated-labelled lectins UEA, SJA, VVL, STL, LEL, DSL, SNA, MAL-II and RCA. An additional incubation with IRDye-labelled streptavidin was used to detect positive lectin binding at different molecular weights (MW). Glycoproteins were revealed by infrared imaging. (TIF) [file pntd.0004688.s001.tif]

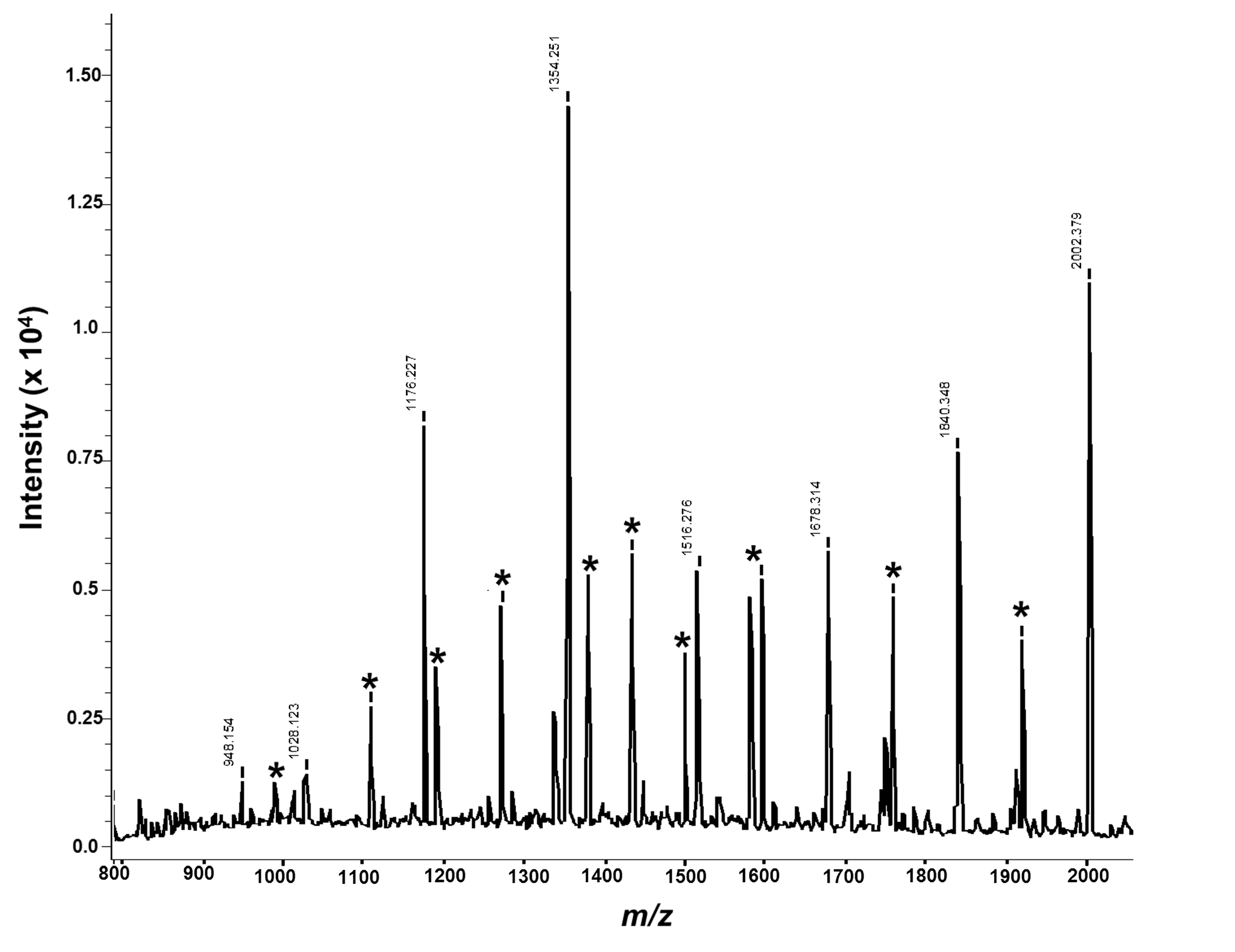

Supplement: S2 Fig — N-glycans released from NEJTeg using PNG-A were labelled with fluorophore 2-aminobenzoic acid (2-AA). MS spectra of AA-labelled N-glycans were acquired by MALDI-TOF-MS. Unidentified peaks are represented with an asterisk (*). (TIF) [file pntd.0004688.s002.tif]

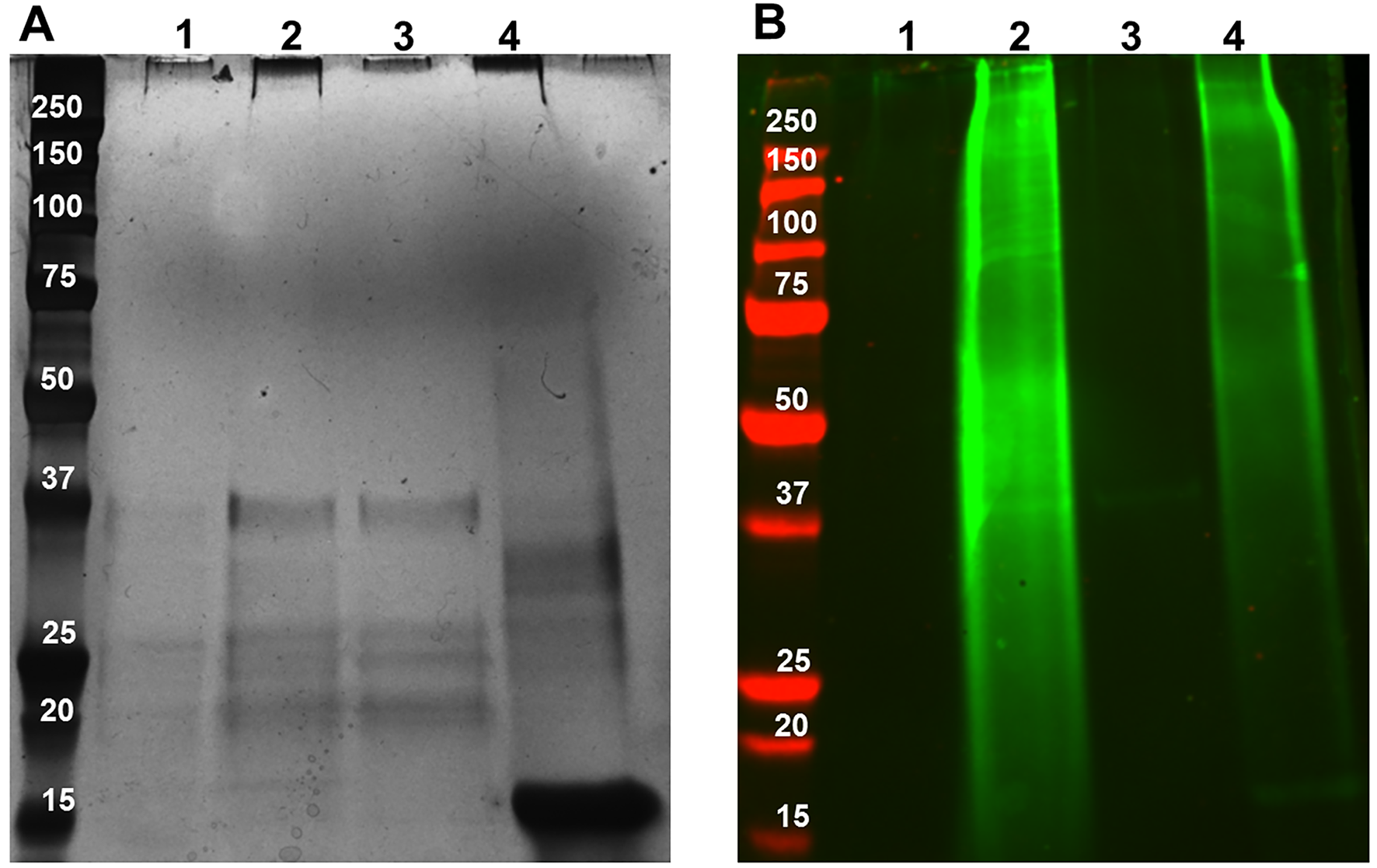

Supplement: S3 Fig — NEJTeg (1) was employed for glycoprotein biotinylation. Biotinylated-NEJTeg (B-NEJTeg) (2) was used for biotin-avidin affinity chromatography, separating avidin-unbound-NEJTeg (A-UB-NEJTeg (3) and the avidin-bound-NEJTeg (A-BB-NEJTeg) (4) fractions. (A) SDS-PAGE and silver staining was used to detect the protein profiles of the four preparations. (B) FITC labelled-streptavidin-incubated membrane was used to confirm correct glycoprotein biotinylation of B-NEJTeg and correct biotin-avidin affinity chromatography separation. (TIF) [file pntd.0004688.s003.tif]
