# Supplementary material for: Tegument Glycoproteins and Cathepsins of Newly Excysted Juvenile Fasciola hepatica Carry Mannosidic and Paucimannosidic N-glycans
Source: PLoS Negl Trop Dis. 2016 May 3;10(5):e0004688. doi: 10.1371/journal.pntd.0004688 (PMC4854454; doi:10.1371/journal.pntd.0004688)
Supplement: S1 File — Multiple sequence alignment of FhCB (BN1106_s5100B000033), FhCB1 (BN1106_s6570B000050), FhCB2 (BN1106_s4482B000044) and FhCB3 (BN1106_s6570B000051) and ProCB2 (gi|27526823) were performed using Clustal Omega. Sequences are ordered according to aligned. Differences in the region containing the N-glycosylation site are highlighted in yellow. Consensus N-glycosylation is underlined. (PDF) [file pntd.0004688.s004.pdf]

S1 File. Multiple sequence alignment of the *F. hepatica* NEJ cathepsins. Multiple sequence alignment of the FhCB (BN1106_s5100B000033), FhCB1 (BN1106_s6570B000050), FhCB2 (BN1106_s4482B000044) and FhCB3 (BN1106_s6570B000051) and ProCB2 (gi|27526823) were performed using Clustal Omega. Sequences are ordered according to aligned. Differences in the region containing the *N*-glycosylation site are highlighted in yellow. Consensus *N*-glycosylation is underlined.

```
BN1106_s5100B000033      ------------------------------------------------------------
BN1106_s4482B000044      MNWLIVFAIIAVVQAKPNHKPQFEAFSDELIRFVNEESGASWKAARSTRFSNVDHFKLHL
BN1106_s6570B000051      MSWLLIFAAIVVAQAKPNYKRQFEPFSDELIHYINEESGASWKAAPSTRFNNIDQVKQNL
gi|27526823              ----------VVVQAAPNEKPQFEPFSDELIHYINEKSGASWKAAPSSRFINIEHFKQHL
BN1106_s6570B000050      MSWLLIFVAIVVVQAAPNEKPQFEPFSDELIHYINEKSGASWKAAPSSRFINIEHFKQHL

BN1106_s5100B000033      ------------------------------------------------------------
BN1106_s4482B000044      GALSETPEERNALRPTIKHDISKNDLPESFDARSQWPQCWTISEIRDQASCGSCWVIANE
BN1106_s6570B000051      GVLEETPEDRNTQRQTVRYSVSENDLPESFDARQKWANCPSISEIRDQSSCSSCWAVSSA
gi|27526823              GLLEETPEERQTRRPTVRYNVSDNDLPESFDAREKWPLCRSIRQIPDQSSCGSCWAVAGV
BN1106_s6570B000050      GLLEETPEERQTRRPTVKYNVSENDLPESFDAREKWP-CRSIRQIPDQSSCGSCWAVAGV

BN1106_s5100B000033      ------------------------------------------------------------
BN1106_s4482B000044      HSTAEKEEIGQIYCIVRHAQILWHRERESFVLLLS--SYHRS-------GASWKAARSTR
BN1106_s6570B000051      SAITDR------ICIHSNGQKKP---RLSAIDIVSCCAYCGYGCNGGIPAMSWDYWTREG
gi|27526823              GAMSDR------VCIHSNGMMQP---ELSAIDLVSCCSYCGNGCQGGSPPAAWDYWWRNG
BN1106_s6570B000050      GAMSDR------VCIHSNGMMQP---ELSAIDLVSCCSYCGNGCQGGSPPAAWDYWWRNG

BN1106_s5100B000033      MNRLLA-------------------FAVIAVVQA----TPSSKTRFDT-----YSD--QL
BN1106_s4482B000044      FSNVDHFKLHLGALSETPEE-RNALRPTIKHDISKNDLPESFDARSQWPQCWTISEIRDQ
BN1106_s6570B000051      --------VVTGGTLENPTGCLPYPFPKCSHGVVTPGLPPCPRDIYPTPKCEKKCH--AG
gi|27526823              --------IVTGGTLENPTGCLPYPFPQCRHP----------------------------
BN1106_s6570B000050      --------IVTGGTLENPTGCLPYPFPQCRHP----------------------------

BN1106_s5100B000033      IHYVNEEAFGAVEAMSDRVCIHSNGQMKPHLSARDLTSCCRTCGFGCKGGYPALAWDYWE
BN1106_s4482B000044      ASCGSCWATAAASAMSDRVCIHSNGQMRPRLAAADPLSCCTYCGQGCRGGYPPKAWDYWM
BN1106_s6570B000051      YNKTTVPAISSASAITDRICIHSNGQKKPRLSAIDIVSCCAYCGYGCNGGIPAMSWDYWT
gi|27526823              ------------------------------------------------------------
BN1106_s6570B000050      ------------------------------------------------------------

BN1106_s5100B000033      SDGIVTGGSLEEQTGCAPYPFPKCSHYESSK-----------------------------
BN1106_s4482B000044      REGIVTGGTWENRTGCQPWMFTKCDHVGDSR-----------------------------
BN1106_s6570B000051      REGVVTGGTLENPTGCLPYPFPKCSHGVVTPGLPPCPRDIYPTPKCEKKCHAGYNKTYEQ
gi|27526823              ---------------------------GSRSQ----------------------------
BN1106_s6570B000050      ---------------------------GSRSQ----------------------------

BN1106_s5100B000033      ------------------------------------------------------------
BN1106_s4482B000044      ------------------------------------------------------------
BN1106_s6570B000051      DKVKGKSSYNVGEQETDIMMEIMKNGPVDGIFYMFEDFLVYXGCNGGIPAMSWDYWTREG
gi|27526823              ------------------------------------------------------------
BN1106_s6570B000050      ------------------------------------------------------------

BN1106_s5100B000033      ----------------------------GLKPCPDELYPTPTCEMKCQAGYNKTYDEDKF
BN1106_s4482B000044      ----------------------------KYSRCPHYTYPTPPCARACQTGYNKTYEQDKF
BN1106_s6570B000051      VVTGGTLENPTGCLPYPFPKCSHGVVTPGLPPCPRDIYPTPKCEKKCHAGYNKTYEQDKV
gi|27526823              -----------------------------LNPCPRYTYPTPSCYPYCQAGYDKTYEKDKV
BN1106_s6570B000050      -----------------------------LNPCP--------------------------

BN1106_s5100B000033      YGNSSYNVEKSEKNIMYEIMKNGPVEATLTVYEDFVVYKSGIYHHVRSRLMGGHAVRILG
BN1106_s4482B000044      YGNSSYNVGEHESYIMQEIMKNGPVEVTFAIFQDFGVYRSGIYHHVAGKFIGRHAVRMIG
BN1106_s6570B000051      KGKSSYNVGEQETDIMMEIMKNGPVDGIFYMFEDFLVYKSGIYHYTTGRLVGGHAIRVIG
gi|27526823              YGKTSYNVDRHEYTIMEEIMKNGPVEAGFIVYTDFAVYKSGIYHHVSGRYAGKHAIRIIG
BN1106_s6570B000050      -GKTSYNVDRNEYTIMQEIMKNGPVEAGFIVYTDFAVYKSGIYHHVSGRYAGKHAIRIIG

BN1106_s5100B000033      WGVENGVKYWLVANSWNEEWGENGYFRIRRGINECGIESEVTAGMPLL--------
BN1106_s4482B000044      WGVENGVNYWLMANSWNEEWGENGYFRMVRGRNECGIESEVVAGMPRL--------
BN1106_s6570B000051      WGVENGVKYWLIANSWNEGWGEKGYFRMRRGNNECGIEARINAGLP----------
gi|27526823              WGVENGVKYWLTANSWNVGWGENGYFRILRGTDECRIESIVVAGMPRLQKNITNHH
BN1106_s6570B000050      WGVENGVKYWLIANSWNVGWGENGYFRMLRGTDECRIESIVVAGMPRLQKNITNRH
```
