# Supplementary material for: Tegument Glycoproteins and Cathepsins of Newly Excysted Juvenile Fasciola hepatica Carry Mannosidic and Paucimannosidic N-glycans
Source: PLoS Negl Trop Dis. 2016 May 3;10(5):e0004688. doi: 10.1371/journal.pntd.0004688 (PMC4854454; doi:10.1371/journal.pntd.0004688)
Supplement: S1 Table — (PDF) [file pntd.0004688.s005.pdf]

S1 Table. Comparison of the protein amino acid sequence of the FhCBs to gi|27526823

Protein	Identifier	Residue Overlap	Identity (%)	Similarity (%)	Gaps (%)	Score
Novel Partial FhCB	BN1106_s5100B000033	224	67.9	74.6	0	844
FhCB1	BN1106_s6570B000050	337	89.6	91.1	8.3	1560
FhCB2	BN1106_s4482B000044	303	66.3	78.9	0	1178
FhCB3	BN1106_s6570B000051	215	69.8	80	0	852

Pairwise alignment settings: EBLOSUM62 matrix, gap opening penalty = 14, gap extension penalty = 4
