# Supplementary material for: Tegument Glycoproteins and Cathepsins of Newly Excysted Juvenile Fasciola hepatica Carry Mannosidic and Paucimannosidic N-glycans
Source: PLoS Negl Trop Dis. 2016 May 3;10(5):e0004688. doi: 10.1371/journal.pntd.0004688 (PMC4854454; doi:10.1371/journal.pntd.0004688)
Supplement: S2 Table — (PDF) [file pntd.0004688.s006.pdf]

S2 Table. Annotation of the additional glycopeptides identified in NEJTeg at bands 1, 2, 3 and 4 that did not correspond to the cathepsins.

Peptide *m/z* $[M+H]^+$	Glycan CID-MS	Bands
2561.2	F1H3N2	2
2560.4	F1H3N2	1
2544.8	F1H3N2	1
2269.2	F1H2N2	1
2269.1	H2N2	1
2095.2	F1H2N2	1
2059.2	F1H2N2	2
2058.9	F1H2N2	2
1869	F1H3N2	1
1868.1	F1H3N2	2
1854	F1H3N2	1
1807.2	H5N2	4
1807.1	H4N2	4
1807.1	H3N2	4
1788.2	H5N2	1, 2
1788.2	H4N2	1, 2
1788.2	H3N2	1
1787.9	H2N2	1
1737.8	H5N2	4
1631.9	H4N2	1
1352	H6N2	4
1340	H5N2	2
1339.7	H5N2	3
1317.8	H5N2	3
1317.8	H4N2	3
1268	H9N2	1, 3, 4
1268	H8N2	3, 4
1252.1	H9N2	1, 3, 4
964.8	F1H5N2	1
964.8	H5N2	1
964.8	F1H4N2	1, 2, 3
964.8	F1H3N2	1, 2, 3, 4
896.8	H5N2	2
860.8	F1H3N2	2
733.8	H5N2	1, 4
733.8	H4N2	1
733.6	H5N2	2
733.6	F1H4N2	1
733.6	H4N2	2, 3, 4
733.6	F1H3N2	2
733.6	H3N2	1, 2, 3, 4
733.6	F1H2N2	1, 2, 3
733.6	H2N2	1
